# A systems biology model of the regulatory network in *Populus *leaves reveals interacting regulators and conserved regulation

**DOI:** 10.1186/1471-2229-11-13

**Published:** 2011-01-13

**Authors:** Nathaniel Robert Street, Stefan Jansson, Torgeir R Hvidsten

**Affiliations:** 1 Umeå Plant Science Centre, Department of Plant Physiology, Umeå University, 901 87 Umeå, Sweden; 2 Computational Life Science Cluster (CLiC), Umeå University, 901 87 Umeå, Sweden

## Abstract

**Background:**

Green plant leaves have always fascinated biologists as hosts for photosynthesis and providers of basic energy to many food webs. Today, comprehensive databases of gene expression data enable us to apply increasingly more advanced computational methods for reverse-engineering the regulatory network of leaves, and to begin to understand the gene interactions underlying complex emergent properties related to stress-response and development. These new systems biology methods are now also being applied to organisms such as *Populus*, a woody perennial tree, in order to understand the specific characteristics of these species.

**Results:**

We present a systems biology model of the regulatory network of *Populus *leaves. The network is reverse-engineered from promoter information and expression profiles of leaf-specific genes measured over a large set of conditions related to stress and developmental. The network model incorporates interactions between regulators, such as synergistic and competitive relationships, by evaluating increasingly more complex regulatory mechanisms, and is therefore able to identify new regulators of leaf development not found by traditional genomics methods based on pair-wise expression similarity. The approach is shown to explain available gene function information and to provide robust prediction of expression levels in new data. We also use the predictive capability of the model to identify condition-specific regulation as well as conserved regulation between *Populus *and *Arabidopsis*.

**Conclusions:**

We outline a computationally inferred model of the regulatory network of *Populus *leaves, and show how treating genes as interacting, rather than individual, entities identifies new regulators compared to traditional genomics analysis. Although systems biology models should be used with care considering the complexity of regulatory programs and the limitations of current genomics data, methods describing interactions can provide hypotheses about the underlying cause of emergent properties and are needed if we are to identify target genes other than those constituting the "low hanging fruit" of genomic analysis.

## Background

Biologists have long been fascinated by the green plant leaf and have tried to understand how leaves are born, live and die. In the last decades, several new approaches to study the structure and function of leaves have emerged: Molecular biology and molecular genetics have, for example, enabled identification of genes that regulate the primary function of the leaf - photosynthesis - and leaf development has been understood in much greater detail; high through-put transcriptomics has identified additional factors influencing leaf function, but traditional transcriptome analyses typically reduces the problem of finding key regulators to detecting differentially expressed genes or computing pair-wise similarity between targets and putative regulators (e.g. hierarchical clustering or co-expression networks). In contrast, systems biology analysis of transcriptional programs treats genes as interacting rather than isolated entities. Thus these methods can begin to understand how so-called emergent properties such as complex phenotypes arise from interacting genes. Whether this can be seen as taking a holistic rather than a reductionistic approach to science has generated quite some debate [1,2], but systems biology methods account for synergistic and competitive effects between regulators that individually could have low similarity to the target. Methods for reverseengineering the transcriptional network from collections of gene expression data have been pioneered on single-cell organisms, but have increasingly been applied to higher order organisms [3] including plants [4,5] where applications of systems biology methods are now emerging. Most systems biology studies have - not surprisingly - utilized using "THE model plant" *Arabidopsis thaliana*, where large transcriptomics programs have generated adequate quantities of high-quality data to enable systems analysis [6]. For example, Carerra *et al. *[4] modeled the transcriptional network of *Arabidopsis *and identified plant-specific properties such as high connectivity between genes involved in response and adaptation to changing environments. However, not all aspects of plant biology can be studied in *Arabidopsis*, which in many respects is a rather atypical plant. Indeed, it was not selected as a model system due to its physiological and ecological qualities, but rather for its suitability for genetic and genomic studies. Therefore, it is important to perform parallel studies in plants with other characteristics, as well as developing the methods to allow data from the *Arabidopsis *system to inform studies in other organisms.

One rapidly emerging plant model system is *Populus *[7]; it's interesting biology (a woody perennial) and the access to a sequenced genome [8] represent an attractive combination. Correspondingly, more advanced data analyses approaches are now being applied in *Populus*. *Populus *provides an attractive model system for studies of leaf biology. For example, Sjödin *et al. *[9] exploited the fact that mature aspen (*Populus tremula*) in boreal regions have the rather unique property that all leaves emerge simultaneously from overwintering buds. This provides a synchronized system, resulting in a full temporal separation of the leaf developmental stages and subsequent acclimation that could be exploited using transcriptomics. Access to a centralized repository of much of the *Populus *cDNA microarray data [10] and databases for the analysis of gene expression - and other - data [11] substantially facilitates the ability to perform systems biology studies. For example, Grönlund *et al. *[12] induced a co-expression network revealing modular architecture explaining gene function and tissue-specific expression; Street *et al. *[13] identified co-expression networks across a large collection of leaf transcriptomics data and found that some network hubs have existing functional evidence in *Arabidopsis*; Quesada *et al. *[14] performed a comparative analysis of the transcriptomes of *Populus *and *Arabidopsis*, and found evidence of extensive remodeling of the transcriptional network, although some essential functions showed little divergence. A few studies have also integrated promoter information to study regulatory control in *Populus*. Shi *et al. *[15] identified combinations of xylem-specific motifs in *Populus *promoters. Another study inferred transcriptional networks in xylem, leaves, and roots, and showed that genes with conserved regulation across tissues are primarily *cis*-regulated, while genes with tissue-specific regulation are often *trans*-regulated [16]. All these studies are essentially co-expression networks that visualize expression similarity between pairs of genes, but do not infer complex interactions.

Network inference methods using expression data can be divided into those that aim to model the general influence that genes have on the expression of other genes (gene networks) [17,18] and methods that aim to model the physical interaction between transcription factors and the regulated genes (gene regulatory networks) [19]. Both approaches employ common network inference methods (see e.g. [20-22]), but those that infer gene regulatory networks also typically integrate motif finding and detection of transcriptional modules [23,24]. Approaches that describe how the regulatory genome orchestrates dynamic gene expression has developed from Pilpel *et al. *[25], who showed that yeast genes sharing pairs of binding sites in their promoters were significantly more likely to be co-expressed than genes sharing only single binding sites, to various machine learning methods that identify modules of co-expressed genes with common motif patterns in their promoters (so-called *cis*-transcriptional modules)[26-34].

Here we apply a network inference method combining promoter information and expression data to describe the transcriptional network in *Populus *leaves. Our aims were (1) to detect regulatory hubs in leaves, (2) to describe conservation of transcriptional regulation within *Populus *and between *Populus *and *Arabidopsis*, and (3) to understand the regulatory complexity in leaves by comparing systems biology and traditional bioinformatics as methods for detecting target genes for further analysis. This study goes beyond previous meta-analyses of *Populus *transcriptome data by taking into account synergistic and competitive interactions between regulators, and by systematically integrating the regulatory genome and the transcriptome to infer networks. We show that our network is robust, explains available gene function information and generalizes to new expression data in both *Populus *and *Arabidopsis*. We identify the main regulators of primary processes in leaves, and show how some of these have regulatory partners orchestrating expression either in a synergistic or competitive manner. Such interactions are not considered by pair-wise similarity methods, and thus several of the regulators predicted here would not have been identified by traditional approaches.

## Results

**Figure 1:**
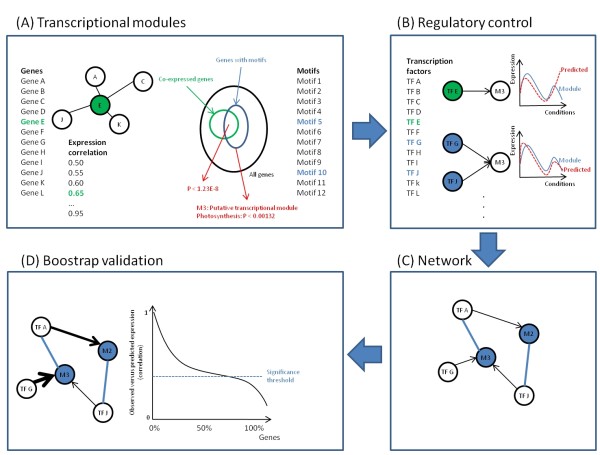
**Method overview**. (A) Transcriptional modules were inferred by searching for motif combinations that were overrepresented in a set of co-expressed genes. Co-expression was defined by a correlation threshold to a central gene, and an exhaustive search was conducted with all genes as centers and applying all thresholds. (B) The regulatory control of each module was inferred by iteratively trying more complex combinations of transcription factors, and stopping when no significant improvement in correlation between observed and predicted expression could be observed. (C) A network was constructed based on the modules and their best transcription factor combinations. (D) The network was validated statistically by bootstrap analysis to test the stability and predictive capabilities.

We inferred the regulatory network of a collection of 562 leaf-specific *Populus *genes with quantified transcription profiles across 465 samples in various experiments such as leaf primordial, budset, biotic infection and drought stress [13] (expression data available in Additional file 1). The approach employed two separate steps to construct the network (Figure 1): First, we discovered a set of representative transcriptional modules containing co-expressed genes with evidence of co-regulation in their promoters. Second, we inferred the most likely regulators (transcription factors) of each module based on gene expression predictability. Thus our model is based on the simple assumption that genes regulated by the same transcription factors should exhibit similar expression profiles across different condition and contain common sequence motifs in their promoters.

### Discovered transcriptional modules reflect important processes in leaves

**Figure 2:**
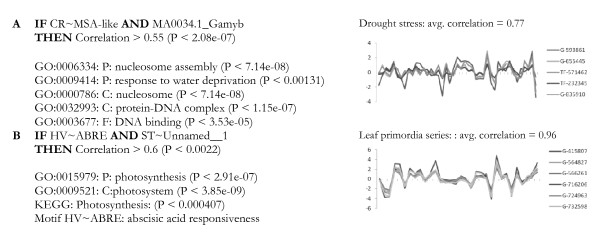
**Example transcriptional modules**. (A, B) Modules are written as IF-THEN rules indicating (causal) relationships between motifs and co-expression. Significant functional annotations are listed below the rules and expression profiles of the co-expressed genes in the modules are plotted for one relevant experimental study.

Putative modules were defined as co-expression genes that could be predicted from sequence motifs in promoters. Significant co-expression was required across all 465 conditions for at least five genes. A large number of overlapping modules were initially induced to capture the rich dynamics of the system. These were then set to compete against each other in an algorithm that produced a final representative library of 38 modules covering 477 genes. Figure 2 shows two examples of these transcriptional modules, while all 38 modules are displayed in Additional file 2. The first module (Figure 2A) contains all genes with the two motifs CR~MSA-like and MA0034.1_Gamyb in their promoters. These motifs were over-represented in co-expressed genes (P < 2.08e-07, expression correlation to the centroid-gene above 0.55). Over-represented functional annotations indicate a role in drought stress and nucleosome assembly. Indeed, a high expression correlation can be observed for these genes in the drought stress experiment (average pair-wise correlation of 0.77). The second example module (Figure 2A) exhibits high expression similarity in the leaf primordial experiment (average pair-wise correlation of 0.96) and annotations indicate a role in photosynthesis. Interestingly, one of the two motifs (HV~ABRE) is a known abscisic acid (ABA) response element, with ABA having a role in many plant developmental processes.

**Figure 3:**
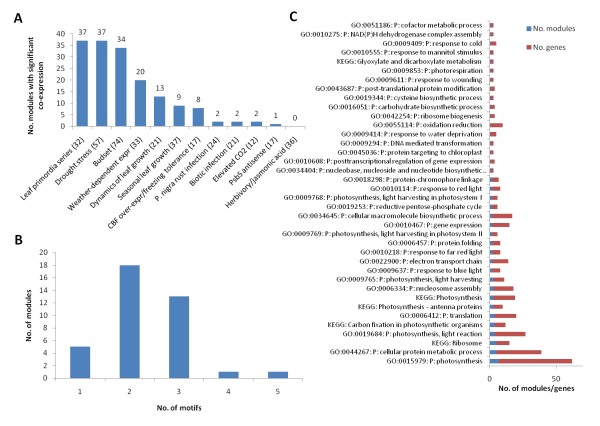
**Transcriptional modules**. (A) The number of modules with significant expression correlation within the different experimental studies. (B) The distribution of modules over different numbers of sequence motifs in their predicted cis-regulatory mechanism. (C) The distribution of modules and genes over functional annotations. The data is only based on annotations statistically over-represented in at least one module, and comprise annotations from Gene Ontology (P: Biological process) and KEGG.

Most modules were significantly co-expressed within developmental processes such as leaf primordial and budset, while only a few modules were co-expressed in stress responses such as biotic infection and elevated [CO_2_] (Figure 3A). Since the expression data are measured by two-channel microarrays, where stress-experiments typically used normal conditions as reference, this indicates that these stress-conditions activate rather different regulatory responses than do development. A notable exception is drought stress, where all but one module exhibit significant co-expression, indicating that drought affects leaf development through these same modules. Interestingly, all of the three modules with a role in nucleosome assembly (e.g. Figure 2A) belong to the very small number of modules with a significant co-expression in stress. The relationship between nucleosome organization and stress has also been reported by others [35] and may indicate a role for epigenetic modifications in response to stress.

One of the goals of this study was to investigate regulatory complexity. Interesting, very few of the discovered modules are associated with only one sequence motif (Figure 3B). Typically two or three motifs were required to find a significant correspondence between motifs and co-expression, indicating a complex relationship between observed expression and the regulatory genome. To evaluate the biological significance of the discovered modules, and their suggested regulatory control, we used functional annotations from Gene Ontology and KEGG. In general, 71% of the modules had some evidence of biological relevance in terms of over-represented Gene Ontology annotations (23 modules) and KEGG annotations (16 modules). Many of these were related to photosynthesis and ribosomal activity, and thus of relevance to leaf development (Figure 3C). Since all genes in this study were leaf-specific with a corresponding over-representation of leaf-specific annotations [13], one could argue that any division of these genes into modules would produce relevant annotations. However, in our statistical tests we used only the leaf-specific genes, not the whole genome, as background to avoid that typical leaf-functions show up as significant just because of the bias in the dataset. Hence, the large fraction of significant modules indicates that our division into modules based on common motifs and co-expression is indeed relevant. This was also confirmed by randomization experiments, which invariably resulted in modules with considerably lower significance than reported here.

### Regulatory network indicates complex regulations

**Figure 4:**
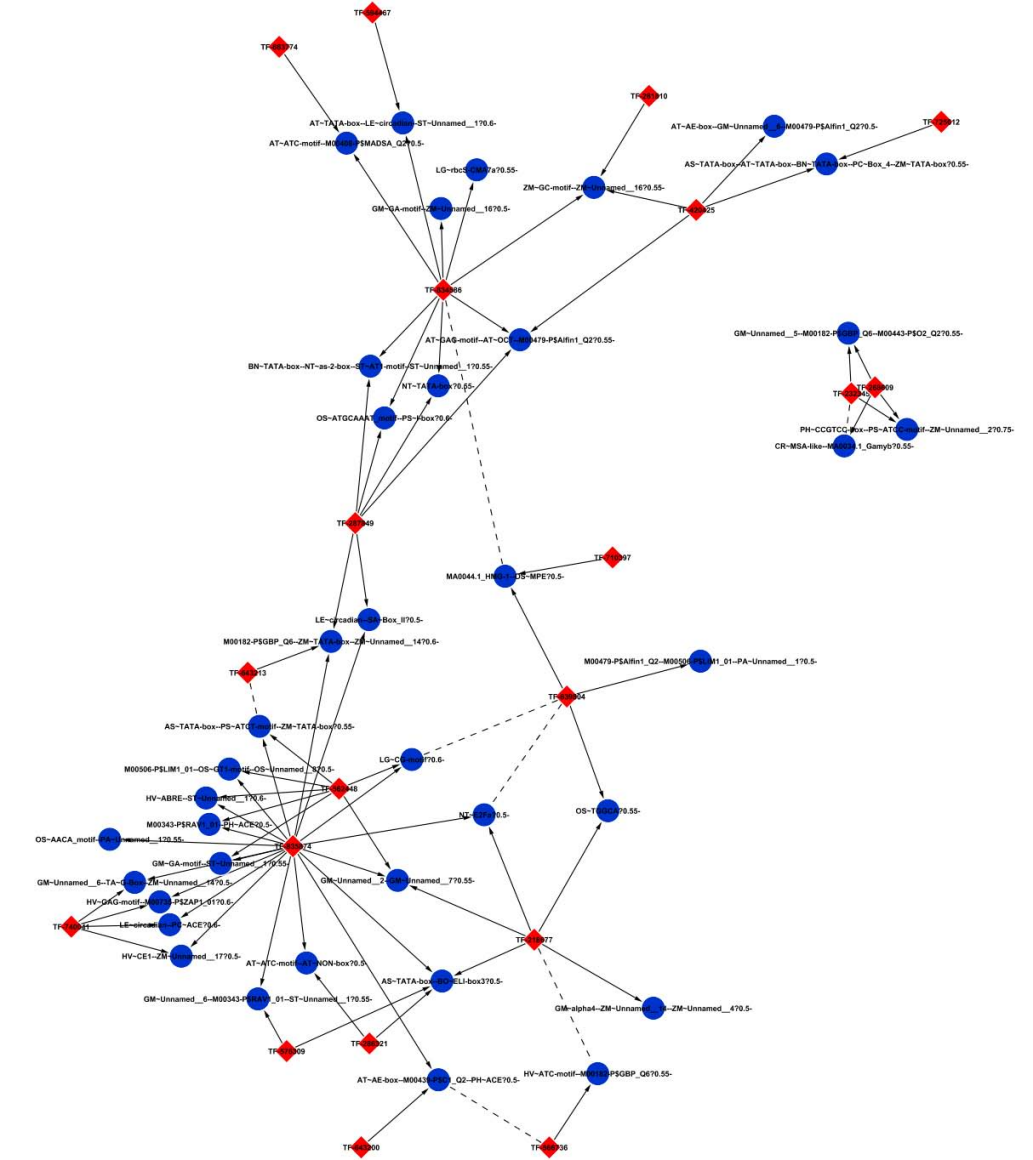
**The transcriptional network of *Populus *leaves**. Regulators (transcription factors) are red diamonds, while transcriptional modules are blue circles.

**Figure 5:**
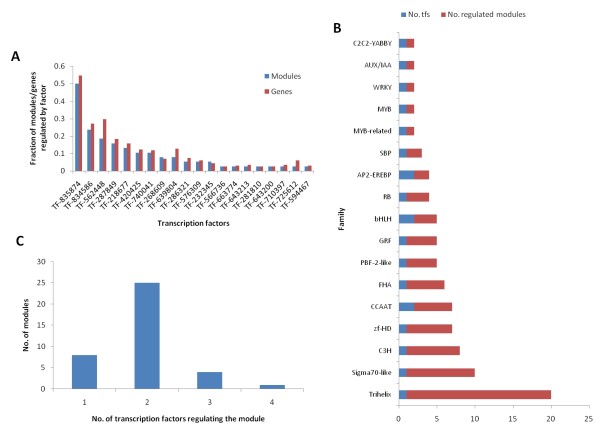
**Network statistics**. (A) The fraction of the total number of modules/genes regulated by each transcription factor follows a power law (the parameters of the fit *ax^b ^*is *a *= 0.62, *b *= -1.1 for modules (R^2 ^= 0.95) and *a *= 0.78, *b *= -1.1 for genes (R^2 ^= 0.95)) (B) The number of transcription factors regulating each module (in-degree) follows a normal-like distribution. (C) Transcription factor families represented in the network.

A regulatory network was inferred by applying regression models to predict the expression of genes in the transcriptional modules from the expression of sets of possible regulators (i.e. transcription factors). The regression models increasingly included more transcription factors until the prediction performance of the more complex model (e.g. three transcription factors) did not significantly improve on the simpler model (i.e. two transcription factors). A network was then drawn based on the best regulators of each module (Figure 4 Additional file 3 and 4). The method allowed us to identify the regulatory hubs of the leaf transcriptional program. As in most biological networks, we observe a few hubs regulating many modules while most transcription factors only regulated a few modules (Figure 5A, B). A particularly strong hub was the transcription factor with protein id 835874. The closest homolog in *Arabidopsis *is ASIL1 (AT3G24490.1). This factor belongs to the Trihelix family of plant-specific transcriptional activators. In our network, it is predicted to be involved in the regulation of all 55 photosynthesis genes that are overrepresented in transcriptional modules (P < 7.08e-07). Table 1 contains a full list of transcription factors predicted to have a regulatory role in *Populus *leaves.

**Table 1:** Predicted regulators of the *Populus *leaf transcriptional program.

	*Arabidopsis*		
Transcription factors	Closest homologue	Functional information	Modules (genes) regulated
835874	ASIL1 (AT3G24490.1)	trihelix family	19/15 (111/91)
834586	SIG1 (AT1G08540.1)	subunit of chloroplast RNA polymerase, response to red and blue light	9/8 (55/50)
562448	K24M9.13(AT3G18640.1)	zinc ion binding	**7/0 (60/0)**
287849	ATHB22 (AT4G24660.1)	embryonic development ending in seed dormancy abscisic acid biosynthetic process, response to water deprivation, heat and osmotic	**6/0 (37/0)**
218677	ABA1 (AT5G67030.1)	stress, xanthophylls biosynthetic process, sugar mediated signaling pathway, response to red light	5/2 (32/12)
420425	ATWHY3 (AT2G02740.2)	defense response	4/3 (25/21)
740041	ATGRF2 (AT4G37740.1)	leaf development	**4/0 (24/0)**
268609	HTA7 (AT5G27670.1)	histone H2A protein, nucleosome assembly	3/3 (14/14)
639804	ATRBR1 (AT3G12280.1)	regulates cell growth, nuclear division and stem cell maintenance	3/5 (26/39)
286321	SPL8 (AT1G02065.1)	megasporogenesis, microsporogenesis	**2/0 (15/0)**
576309	T10K17.10 (AT3G57800.2)	basic helix-loop-helix (bHLH) family	2/1 (12/5)
232345	HTA10 (AT1G51060.1)	histone H2A protein, nucleosome assembly	**2/0 (9/0)**
566736	T6L1.10 (AT1G68920.3)	basic helix-loop-helix (bHLH) family regulation of flower development, meristem	1/1 **(5/5)**
663774	YAB1 (AT2G45190.1)	structural organization, abaxial cell fate specification	**1/0 (6/0)**
643213	IAA14 (AT4G14550.1)	response to auxin stimulus, lateral root morphogenesis	**1/0 (7/0)**
281810	ATWRKY44 (AT2G37260.1)	epidermal cell fate specification, seed coat development	**1/0 (5/0)**
643200	ATERF-9 (AT5G44210.1)	ethylene mediated signaling pathway cinnamic acid biosynthetic process,	**1/0 (5/0)**
710397	ATMYB3 (AT1G22640.1)	response to wounding, salt stress and abscisic and salicylic acid stimulus, negative regulation of metabolic process cell death, response to stress, ethylene	**1/0 (7/0)**
725612	ATEBP (AT3G16770.1)	mediated signaling pathway, response to cytokinin stimulus, ethylene stimulus and other organism	**1/0 (12/0)**
594467	ETC1 (AT1G01380.1)	involved in trichome and root hair patterning	**1/0 (6/0)**

*Populus *v1.1 protein ID is given together with information on the closest homologue in *Arabidopsis*. The last column gives the number of modules (and in parenthesis the number of genes) regulated by the factor in our systems biology-based network and in the co-expression network, respectively. Transcription factors in our systems biology-based network that are not in the co-expression network are marked in bold.

Our method of increasingly evaluating more complex regulatory mechanism allowed us to quantify the complexity of the regulation in *Populus *leaves. The distribution of modules over the number of transcription factors in the predicted regulatory mechanism (Figure 5C) roughly follows that of the number of motifs (Figure 3B). Thus, the predictive power of the regulatory mechanisms of most modules benefit significantly from including more than one transcription factor. Both steps in our method predict expression of genes, however, while the module discovery approach finds sequence motifs predictive of gene expression clusters, the network inference approach finds transcription factors predictive of the gene expression in each module. Both approaches are guided by the principle of Occam's razor, that is, that the simplest model explaining the data is the best, and both approaches, as we have seen, result in the same distribution for the number of regulators per module.

**Figure 6:**
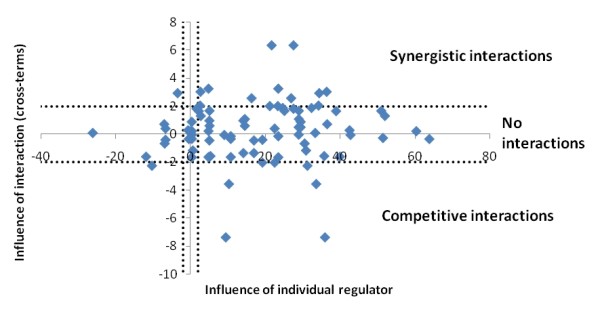
**Regulatory complexity**. The influence of the interaction between each pair of regulators (i.e. the cross-term *β*_12 _in the case of two regulators) is plotted against the influence of each individual regulator (i.e. *β*_1 _and *β*_2 _in the case of two regulators). In order to compare these values independently of the expression intensities of the particular module and transcription factors, we have plotted the T-statistics of the *β*'s rather than their actual values. Statistically significant values are marked by dotted lines.

The regression models describe the expression profiles in modules using the expression profiles of transcription factors. In the case of two regulators, the expression of a module *m *is represented as a weighted sum of the expression of the regulators tf_1_and tf_2_, i.e. m = *β*_0 _+ *β*_1_tf_1 _+ *β*_2_tf_2 _*β*_12_tf_1_tf_2_. Thus, after fitting this model to the available expression data, the values of *β*_1 _and *β*_2 _will reflect the importance of each individual regulator, while the value of *β*_12 _(the cross-term) will reflect the importance of the interaction between the two regulators. If the cross term is close to zero, there is a linear relationship between the module and the regulators, and not necessarily an interaction between the regulators. A positive value of the cross term indicates a synergistic relationship between the regulators, while a negative value indicates a competitive relationship [36]. Figure 6 shows that individual regulators have a strong preference towards positive regulation over negative (88% versus 12%). We also see slightly more synergistic than competitive relationships between regulators (56% versus 44%). Seven modules are governed by statistically significant synergistic interactions, while four modules exhibit competitive regulation (see Additional file 4 for details).

The network is fully connected except for a small sub-network of the three nucleosome assembly modules discussed earlier. One of these modules is shown in Figure 2A, and is predicted to be regulated by 268609 (HTA7, closest homolog AT5G27670.1). This factor is a histone protein with a known role in nucleosome assembly (Table 1). The other two modules are predicted to be regulated by 268609 in concert with 232345 (HTA10, closest homolog AT1G51060.1), also a histone protein with a known role in nucleosome assembly. The protein 232345 is itself a member of the example module from Figure 2A. The fact that we did not allow auto-regulations in our inference method might thus be the reason why this module only has one regulator (i.e. 268609). The two modules associated with both factors are the two modules with the strongest competitive regulatory mechanisms in the network (Figure 6). Both these regulators have a significant individual influence on the expression of the modules, but they also have a highly significant negative cross-term indicating the competitive regulation. Intriguingly, these are the only two modules in the network with a significant co-expression during biotic infection, although they are also co-expressed in a number of other experiments.

### Regulatory network predicts expression in unseen experiments

Bootstrap analysis is often used in computational studies to evaluate the statistical significance of models such as phylogenetic trees [37]. A bootstrap dataset has the same number of genes and conditions as the original data, but with some conditions occurring several time and some conditions not occurring at all (i.e. drawn with replacement). On average, 36.8% of the conditions will not occur in the bootstrap dataset and we refer to this as the *hold-out set*. Our network was validated statistically by first inferring a number of networks from different bootstrap dataset, and then (a) assessing the agreement between these bootstrap networks and the original network (stability) and (b) using the regression models from the bootstrap networks to predict expression values in the hold-out sets (predictive power).

**Figure 7:**
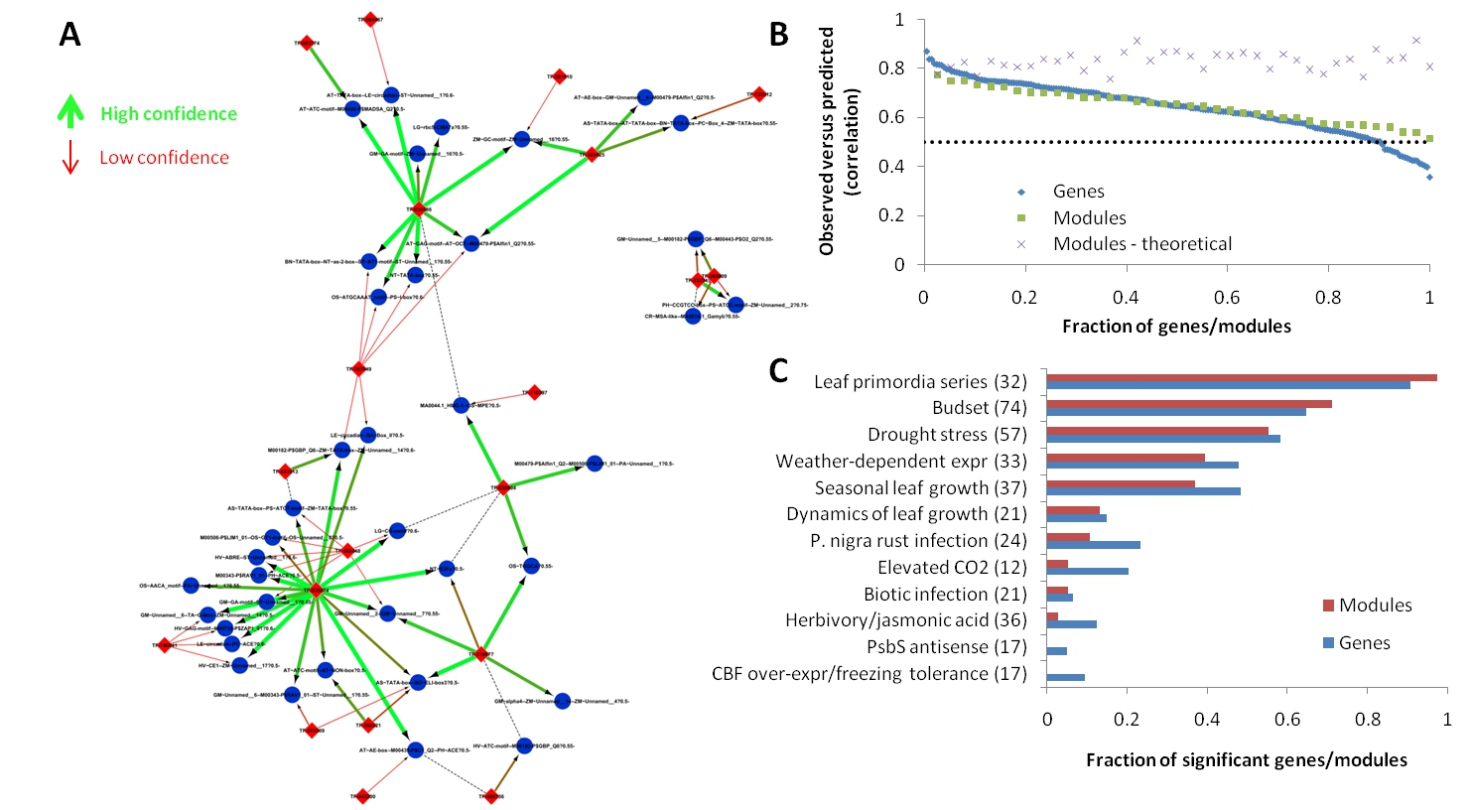
**Bootstrap analyses of the network**. (A) The transcriptional network with edges colored from red to green, and increased thickness, with increasing bootstrap confidence. (B) Correlation between observed and predicted gene expression averaged over experimental conditions not used to infer the bootstrap networks (i.e. the hold-out set). Correlations are shown for individual genes, modules (average correlation for each gene in the module) and a theoretically optimal prediction (predicted expression equal to the average expression profile of the genes in the module). (C) Fraction of genes and modules with a significant correlation between observed and predicted gene expression in each experiment when that experiment was removed before inferring the network.

Most predicted regulations in the network recurred in a majority of the bootstrap networks (43/74 = 0.58). However, about every third regulation had low support (23/74 = 0.31) (Figure 7A). Three hubs (protein ids 562448, 740041 and 287849, see Figure 5) were the sources of 17 of these 23 weak regulations (Figure 7A). They are predicted to co-regulate modules with other, stronger regulators, and typically do not regulate modules by themselves. Thus these predicted regulatory interactions are sensitive to data removal and may only be valid under some experimental conditions.

Our *Populus *network models show a remarkable ability to generalize to unseen conditions, although similar predictive capability has been demonstrated also for other organisms [4,38]. Since we use the expression of a set of transcription factors to predict *one *expression profile per module, the correlation between observed and predicted expression is limited by the degree of expression similarity of genes within modules. Still, all co-expressed genes in modules had a significant correlation between observed and predicted expression when using the bootstrap networks to predict the expression in the hold-out sets (Figure 7B). In fact, 90% of genes, and all the modules, obtained a correlation above 0.5 (the original threshold for including genes in modules). We also held out entire experiments (e.g. budset, biotic infection, etc.) and used the resulting networks to predict the expression values in the missing experiment (Figure 7C). Since few modules have a significant expression similarity within modules in stress responses (Figure 3A), we are naturally unable to predict the expression in these experiments. However, the regulation of the developmental programs, in particular leaf primordia and budset, can be predicted from the other experiments (Figure 7C). This is also true for drought stress, indicating that regulation of drought response corresponds to the regulation of development in that there is a conserved relationship between regulating transcription factors and regulated gene modules. A notable exception is the nucleosome assembly modules from Figure 2A with a role in water deprivation response. This role is confirmed by the fact that the expression profile of this module cannot be predicted without the drought stress dataset (correlation -0.24 versus 0.56 in the bootstrap analysis).

### Several regulatory mechanisms are conserved between *Populus *and *Arabidopsis*

**Figure 8:**
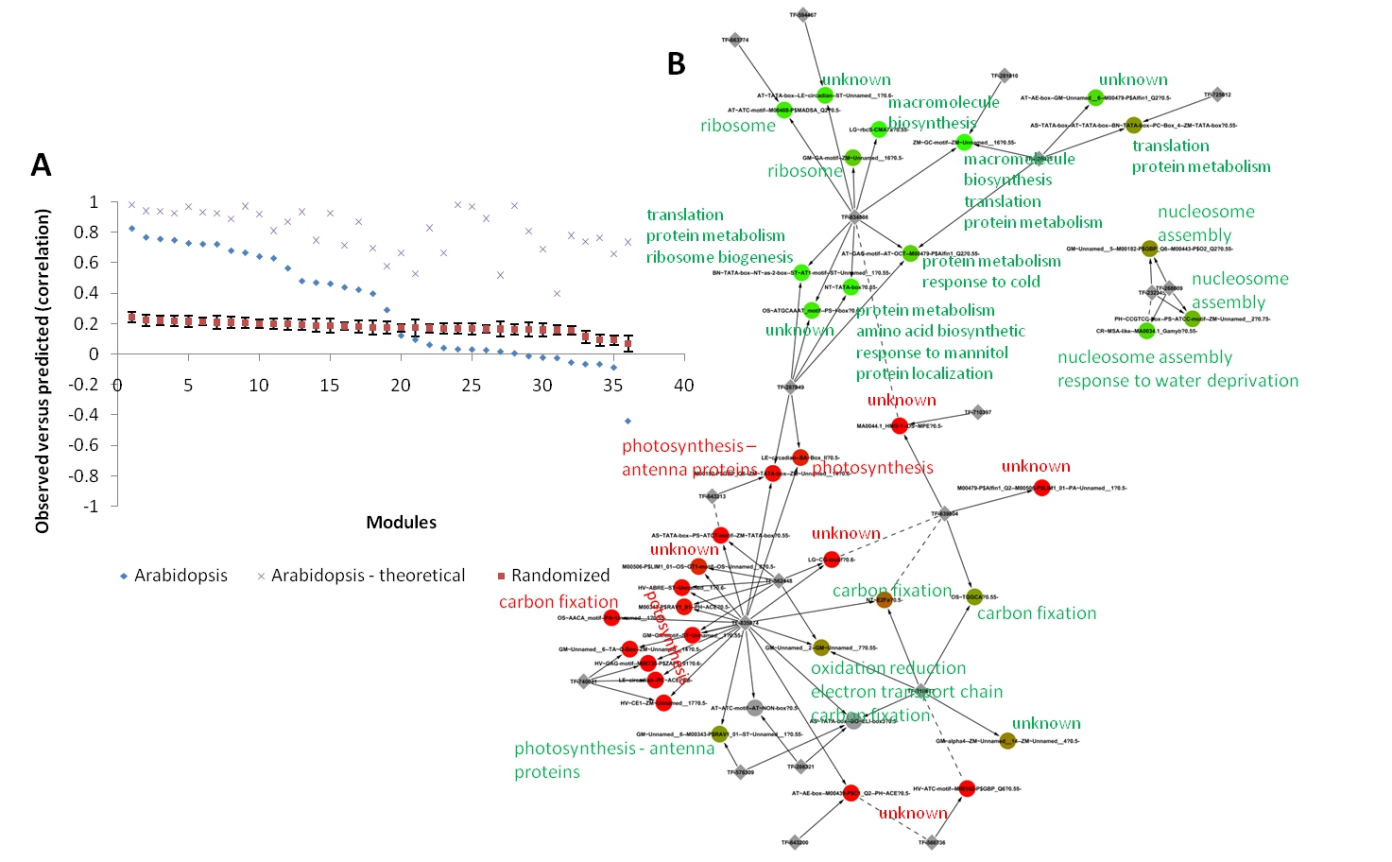
**Comparative genomics**. (A) Correlation between observed and predicted expression of the modules in *Arabidopsis *using the network inferred from *Populus*. The theoretically optimal prediction is also shown and indicates that all modules are predictable in *Arabidopsis*. The randomized curve is based on 1000 runs where the *Arabidopsis *genes are randomly assigned to modules. (B) The regulatory network with modules colored from green (conserved, high correlation) to red (non-conserved, low correlation) based on the expression correlation from (A). Grey modules lack homologues or expression data for their genes or regulators. Modules are labeled with the main functional annotations.

The aim of comparative genomics is usually to investigate the conservation of sequence across different species. However, while proteins have diverged surprisingly little between related species, regulatory networks are believed to evolve much faster [39]. Our predictive approach makes it possible to investigate to what degree regulatory mechanisms of modules inferred from *Populus *are conserved in other plant systems. We applied the regression models from our *Populus *inferred network to predict the expression of closest homologues in *Arabidopsis *using the AtGenExpress developmental conditions [40]. Since we were predicting the expression of *Arabidopsis *genes from the expression of *Arabidopsis *transcription factors, we were not testing the co-expression of these genes between the two plants. Rather, we were testing whether the regulatory mechanism, i.e. the relationship between transcription factors and genes, is conserved. Of the 36 modules with expressed homologues in *Arabidopsis*, 50% showed conservation beyond what would be expected by chance (correlation ≥ 0.40, Figure 8A and Additional file 5). These 18 conserved modules cluster in three distinct parts of the network with functional roles in (1) biosynthesis, protein metabolism and translation, (2) carbon fixation, and (3) nucleosome assembly (Figure 8B). On the other hand, the non-conserved modules are almost exclusively over-represented for photosynthesis genes, showing a clear functional distinction between modules with conserved regulation in *Arabidopsis *and those without. Interestingly, the photosynthesis modules contain co-expressed genes also in *Arabidopsis*, although less so than the modules with conserved regulation (Figure 8A). Thus, the model does not predict that the photosynthesis modules themselves have diverged between *Populus *and *Arabidopsis*, but rather that the regulation of these genes has been rewired. This predicted rewiring of photosynthesis could be explained by the divergence in expression of the hub ASIL1. We also repeated the analysis for *Arabidopsis *expression data observed under abiotic stress (16 modules conserved), biotic stress (6) and various light conditions (4). Thus, the observation that abiotic stress (e.g. drought stress) perturbs our modules to a less degree than biotic stress (e.g. biotic infection) also extends to the *Arabidopsis *data.

Other studies have also investigated the conservation of gene expression across *Populus *and *Arabidopsis*. Quesada *et al. *[14] reported evidence of extensive evolution of gene expression regulation. Street *et al. *[13] identified hub-genes in leaf development, and quantified the fraction of conserved genes to about 60%. Our results seem to imply similar conclusions, although the present study directly identified conserved relationships between transcription factors and gene modules. An interesting question not addressed here is to what degree evolution of gene expression can be explained by divergence in the regulatory regions (promoter sequences) of the two species.

### Systems biology predicts new leaf regulators

Our approach describes interactions between regulators by inferring sets of transcription factors that regulate modules in concert. This systems biology approach differs from traditional analysis such as hierarchical clustering or co-expression networks that only consider pair-wise similarity between the regulator and the regulated genes. To compare these two approaches, we also constructed a co-expression network where each module is regulated by the single transcription factor with the most similar expression to that module (Additional file 6). Table 1 lists all transcription factors in our systems biology-based network, and compares these to the regulators in this reductionistic co-expression network. While the co-expression network identifies 8 transcription factors as regulators of *Populus *leaf transcription, our network includes 20 of the 35 transcription factors in the data. From Figure 6 it was apparent that most collaborative regulations in our network have a master regulator, and this is the regulator typically identified by the co-expression network. Thus, most of the new regulators in our network are due to the fact that collaboration between transcription factors explains more of the expression in modules than single factors. Thus, although the regulations in our network are considerably stronger in terms of prediction power, they rarely exclude the transcription factor found in the co-expression network. However, two modules were predicted by the co-expression network to be regulated by transcription factor 639804 even though this most-similar factor was excluded as a regulator in our network. Somewhat surprisingly, the proposed regulatory mechanisms in these two cases are a weighted sum of two and three transcription factors without a statistically significant synergistic or competitive interaction (i.e. non-significant cross-terms).

## Discussion

One of the aims of systems biology is to model the complex interactions in living cells, describing emerging properties not apparent from studying genes, proteins or metabolites individually. Still, most computational approaches just take pair-wise similarity, not interactions between genes, into account when inferring network from expression data. The reason for this is at least two-fold. First, exploring combinatorics is computationally expensive. For example, there are over 2000 transcription factors in *Populus *giving rise to over 2 million pairs, 1.3 billion triplets, etc. Second, more complex models (e.g. cross-terms in regression models) imply many more parameters that have to be estimated from data (i.e. the *β*'s in regression models). Since we need more observations than model-parameters to avoid over-fitting the models, the number of required observations grows quadraticly with the number of regulators when considering pairs. This *curse of dimensionality *represents a huge obstacle to studying interactions in biological systems. Here, we deal with these problems in several different ways. First, we restrict our study to leaf-specific genes rendering far fewer combinations than an unfiltered whole-genome study. Second, rather than considering all regulators at once, we devised a method that starts with single regulators, and then moves to pairs and higher-order combinations. This provides adequate observations to estimate parameters for each model (365 observations versus only four parameters in the case of two regulators), but because we test so many different models it comes with the risk of finding combinations that obtain high predictive power by change (i.e. over-fitting). We deal with this problem by only increasing model complexity if a statistically significant boost in predictive power is observed on unseen data (cross validation). In the statistical test we used the highly-conservative Bonferroni correction where the initial significance threshold (0.05) was divided by the number of transcription factor combinations tested.

For the leaf-specific genes studied here, the systems biology-based network mostly discovered co-regulators to the transcription factors also identified in the co-expression network. That means that although 11 of 38 modules had regulatory mechanisms with a significant interaction term (cross-term, Figure 6), these regulators also had significant individual contributions of which the strongest is detected by pair-wise similarity. A situation where the cross-term is significant, while the individual contributions are non-significant, is not observed in this data. An example of such a regulation is the logical XOR, that is, the regulated module is up-regulated only if one of the regulators is up-regulated (but not both). Whether such regulations exist in *Populus *leaves cannot be settled from this study considering the limited set of genes included. Interestingly, the interaction term was non-significant in both cases where the best individual regulator was not part of our regulatory mechanism, meaning that the single best regulator was outperformed by a linear combination of other regulators. Such examples demonstrate how systems biology approaches have a better power to dissect regulatory complexity of biological systems than traditional approaches [1,41,42]. They also show that systems biology is able to better model the 'real world' as QTL analysis of quantitative traits typically identifies numerous genetic loci, suggesting the involvement of numerous genes.

A particularly appealing feature of regression-based networks is their ability to predict expression of genes based on the expression of transcription factors. We have used this to quantify the stability and predictive power of the network, but also to study module-conservation between experiments in *Populus *and in *Arabidopsis*. Several interesting predictions were found when studying modules that are co-expressed and correctly predicted using bootstrap networks, but that lose their predictability in particular experiment when these are entirely removed before network inference. We have already mentioned the nucleosome assembly modules that are predicted to be regulated by histone H2A proteins. The drought response profiles in these modules cannot be predicted by networks not trained on drought stress data. Another module (characterized by motifs AS~TATA-box, AT~TATA-box, BN~TATA-box, PC~Box_4 and ZM~TATA-box) was affected by the removal of the budset data and is predicted to be regulated by factor 725612, a known cell death regulator. The module characterized by motif OS~TGGCA looses predictability without the dynamics of leaf growth-dataset, and is predicted to be controlled by a cell growth regulator (protein id 639804). Genes in this module are also over-represented for carbon fixation. Prediction is a central theme in this study, and we strongly believe that predictive models have a lot to offer experimental biology as hypotheses generators.

The complete and correct regulatory network of an organism cannot be reverse-engineered from a limited collection of gene expression data. However, we believe that such models represent a powerful starting point for further analysis as both hypothesis generation and descriptive tools. The hubs in our network (Table 1) thus represent attractive candidates for Chip-seq analysis, functional knock-down studies and regulon engineering. The network we present here only reflects the best regulators of each module. However, behind each module in this network there is a ranked list of regulatory mechanisms (Additional file 4), and as we have seen through bootstrap analysis, the ranking of these lists is not written in stone. In the future one might hope that additional, and higher quality, data (e.g. RNA-Seq) will enable creation of more robust network models that more accurately reflect the underlying biological truth. Obviously, even a perfect network inference method cannot be better than the data it is modeled on (junk in, junk out). Another route to more reliable networks lies in combining computational inference with experimental testing in an iterative modeling approach. Several studies have shown how systematic perturbation of critical pathway components can be used to refine network representations [43,44]. In plants, the *lignin systems*-project is taking this approach to model the lignin biosynthesis pathway [15]http://www.ligninsystems.org. Other sources of information may also be integrated into the network, but were not considered here, including epigenetic signatures such as nucleosome positioning and methylation patterns [45], predicted binding site strength and transcription factor binding site preference [46], and miRNA regulation [47].

## Conclusions

We have outlined a systems biology model of the regulatory network of *Populus *leaves. The approach goes beyond previous analyses of *Populus *transcriptome data by systematically considering interactions between transcription factors, leading us to predict new regulators of leaf development not found by traditional genomics methods. These regulators orchestrate the transcriptional program in a synergistic or competitive manner, and thus constitute non-obvious targets for further analysis. The model is robust when applied to predict expression levels in new data, and reveals conserved and diverged regulation both in different conditions within *Populus *and between *Populus *and *Arabidopsis*.

## Methods

### *Populus *expression data

Street *et al. *[13] identified 562 leaf-specific *Populus *genes that were profiled using *Populus *cDNA microarrays in 465 different experimental conditions (data available in UPSC-BASE [13] and in Additional file 1). These experiments included *budset *(74 conditions), *biotic infection *(21), *weather dependent gene expression *(33), *CBF over express/freezing tolerance *(17), *seasonal leaf growth *(37), *elevated CO2 *(12), *PsbS antisense *(17), *leaf primordia *(32), *dynamics of leaf growth *(21), *P. nigra rust infection *(24), *herbivory/jasmonic acid *(36), *drought stress *(57) and various other conditions (84).

### Sequence motifs and promoters

We created a database of 312 non-redundant plant-related transcription factor binding sites from PlantCare [48], Transfac [49] and JASPAR plantae [50]. From the initial set of 470 motifs, we iteratively identified the two most similar motifs and removed the longest until no pair had a MotifComparison [51] distance bellow 0.3. 2000 bp *Populus *promoters were taken from the PopGenIE online resource http://popgenie.org/[11] and MotifScanner [51] was used to scan these promoters for occurrences of the motifs. MotifScanner was run with a second order background model created from all *Populus *promoters and an *a prior *probability of finding one instance of the motif equal to 0.2. 307 motifs had hits to at least five genes in the leaf expression dataset.

### Transcriptional module discovery

We have previously developed a method for discovering transcriptional modules that uses rule-based machine learning to find combinations of motifs that are predictive of co-expression [29-31]. Here we used this approach to find modules within the leaf experiments. For each gene, we identified all co-expressed genes at different levels of expression similarity and applied the rule learning method to find motif combinations explaining this co-expression pattern. Two genes were deemed co-expressed if their expression profiles had a Spearman correlation coefficient higher than a threshold (calculated based only on the experiments where both genes had measured expression). This threshold was varied from 0.50 to 0.95 in steps of 0.05. Only motif combinations with at least five genes over the co-expression threshold, and no more than 50 genes below the threshold, were considered. P-values for the overlap between genes with the motif combination and co-expressed genes were computed using the hyper-geometric distribution, and only FDR-significant rules (controlled at 0.05) were retained.

Gene function annotations were taken from KEGG [52] and Gene Ontology (GO) [53]. Since GO do not provide annotations for *Populus *genes, we took annotations from the five closest proteins in the GO database with BLAST E-value less than 1E-6 or, if BLAST gave no hits, PSI-BLAST E-value less than 1E-6. Using the hyper-geometric distribution, we computed p-values for all annotations (at all levels in GO) with assignments to at least two co-expressed genes in a module, and retained all FDR-significant annotations (controlled at 0.05). We also performed randomization experiments by randomly shuffling promoters among the genes to create 1000 randomized data sets, and then performing module discovery and annotation analysis of each of these.

### Network inference

We used a least square regression model to infer regulators of each transcriptional module. Here, the expression of a module *m*_*i *_was modeled as the weighted sum of the expression of a set of transcription factors *m*_*i *_= *β*_0 _+ ∑_*j *∈ *R *_*β*_*j*_*t*_*j *_+ ∑_*j, k *∈ *R, j *<*k *_*β*_*jk*_*t*_*j*_*t*_*k*_, where *t_j _*is the transcription factor with index j and R is the set of transcription factor indices. The best regulators of each module were found by estimating the performance of different sets of possible regulators R. Performance was quantified as the correlation between observed (i.e. measured by cDNA microarray) and predicted expression during cross validation (five iterations of 5-fold cross validation). The order of R was iteratively increased from single transcription factors (order 1), to pairs of transcription factors (order 2), etc. The best set of regulators of order *n *was selected as the final regulatory mechanism of the module if no set of regulators of order *n+1 *could predict expression of the module significantly better. Significance was determined by using the Bonferroni corrected p-value (i.e. multiplied by the number of transcription factor combinations tested) calculated using a t-test for the difference between two non-independent Pearson correlations [54]. The expression profile of a module was defined as the concatenation of the expression profiles of each co-expressed gene in the module. The regulatory networks was constructed by using transcription factors and modules as nodes, and drawing an edge between a transcription factor and a module if the transcription factor was part of the best regulatory mechanism for that module.

### Bootstrap analysis

We drew 100 bootstrap datasets from the original 465 conditions in the leaf dataset (i.e. 100 samples of 465 conditions drawn with replacement) and inferred networks from each of these datasets. The regression model of each module was then used to predict the expression in non-sampled conditions for the co-expressed genes in that module. For each gene, predicted expression values from each condition were averaged across the bootstrap samples, and correlation between observed and predicted expression was calculated. The resulting correlation for a gene was thus only calculated for conditions that were not part of at least one bootstrap sample. We also investigated the stability of the regulations by calculating the fraction of bootstrapped networks that contained each edge in the original network.

### Comparative genomics

*Arabidopsis *data was taken from the AtGenExpress resource: development (237 conditions) [40], abiotic stress (298) [55], biotic stress (108) and light (48). We mapped our *Populus *proteins to the closest proteins in *Arabidopsis *as detected by BLAST [11]. We then used regression models trained on the *Populus *expression data to predict expression in *Arabidopsis*.

## Authors' contributions

NS compiled the expression data, the annotations and the motifs, and carried out the motif matching. SJ advised the design and the biological interpretation. TRH designed the study, carried out module discovery and network inference, and drafted the manuscript. All authors participated in discussions, analysis and interpretation, and wrote the manuscript. All authors read and approved the final manuscript.

## Supplementary Material

Additional file 1 **Gene expression data**. The gene expression matrix used in this study. Click here for file

Additional file 2 **Transcriptional modules**. All transcriptional modules in the library with over-represented function information. Click here for file

Additional file 3 **Transcriptional network**. The inferred transcriptional network in text format (ready to be viewed in Cytoscape). Click here for file

Additional file 4 **Predicted regulatory mechanisms**. The regulatory mechanisms predicted for each transcriptional module. Click here for file

Additional file 5 **Conserved transcriptional modules**. Transcriptional modules that have conserved regulation in the *Arabidopsis *developmental data. Click here for file

Additional file 6 **Co-expression network**. The inferred co-expression network based on pair-wise similarity (ready to be viewed in Cytoscape). Click here for file
