# Supplementary material for: A systems biology model of the regulatory network in Populus leaves reveals interacting regulators and conserved regulation
Source: BMC Plant Biol. 2011 Jan 13;11:13. doi: 10.1186/1471-2229-11-13 (PMC3030533; doi:10.1186/1471-2229-11-13)
Supplement: Additional file 2 — Transcriptional modules. All transcriptional modules in the library with over-represented function information. [file 1471-2229-11-13-S2.PDF]

Description:

Line by line description for each module:

- IF-THEN rules: The set of motifs (IF-part, motif names separated by AND) over-represented in genes with similar expression to a central gene (THEN-part, protein id and correlation threshold).
- P-value support in the expression data: Central gene, p-value and statistics (no. genes with expression, no. genes matching the motifs (IF-part), no. genes with similar expression to the central gene above the threshold (THEN-part), no. genes matching both the IF and THEN part)
- Coverage negative genes: genes matching the motifs (IF-part), but have expression similarity below the threshold
- Coverage positive genes : genes matching both the IF and THEN part
- Significance threshold: False discovery rate (FDR) thresholds for over-representation of Gene Ontology term or KEGG categories. Threshold of 0 means nothing is significant.
- Significant functions: function, p-value, statistics (no. genes with a function annotation, no. genes with function annotation among the positive genes, no. genes annotated with the function, no. positive genes annotated with the function), positive and negative genes with the function.

```
-------------------------------------------------------------------------------

IF M00506-P$LIM1_01 AND OS~GT1-motif AND OS~Unnamed__8 THEN G-675692-0.5
G-675692-0.5: 0.001993 (562,43,33,8)
Cov-neg: G-172100 G-234689 G-240319 G-260250 G-269043 G-288961 G-415487 G-566261 G-572354 G-575185 G-593861 G-642306 G-654077 G-655445 G-673777 G-706754 G-712554 G-712747 G-715371 G-715463 G-725114 G-726168 G-737207 G-746197 G-784099 G-802185 G-821599 G-827700 G-829854 G-831371 G-832074 G-832630 G-835242 G-837320 TF-286321
Cov-pos: G-197108 G-229824 G-271570 G-556890 G-675692 G-791055 G-808774 G-827935
Significance threshold GO: 0 (FDR)
Significance threshold KEGG: 0 (FDR)

IF OS~AACA_motif AND PA~Unnamed__1 THEN G-714062-0.55
G-714062-0.55: 1.625397e-06 (562,19,14,6)
Cov-neg: G-206841 G-267999 G-409886 G-410455 G-419541 G-555320 G-567316 G-649116 G-826191 G-828771 G-837320 TF-232345 TF-552123
Cov-pos: G-416054 G-706715 G-714062 G-744163 G-820389 G-822298
Significance threshold GO: 0 (FDR)
Significance threshold KEGG: 0.0431266538657268 (FDR)
KEGG: Carbon fixation in photosynthetic organisms: 0.0431266538657268 (140,4,13,2) (5) Genes: G-567316 G-744163 G-820389

IF GM~GA-motif AND ZM~Unnamed__16 THEN G-816277-0.5
G-816277-0.5: 0.000135 (562,47,46,12)
Cov-neg: G-179495 G-227384 G-228329 G-254402 G-271570 G-282545 G-418525 G-421814 G-424279 G-550669 G-557360 G-558970 G-568201 G-578419 G-646180 G-674736 G-677905 G-717022 G-717926 G-736262 G-746197 G-816237 G-819059 G-822177 G-823815 G-827700 G-827935 G-830089 G-831715 G-832349 G-837468 TF-556378 TF-640573 TF-643871 TF-819435
Cov-pos: G-171713 G-229824 G-254603 G-296649 G-415487 G-416054 G-559187 G-735683 G-744851 G-766070 G-791055 G-816277
Significance threshold GO: 0.000450412413352255 (FDR)
GO:0000313: C: organellar ribosome: 0.000450412413352255 (537,12,8,3) (219) Genes: G-229824 G-559187 G-766070
Significance threshold KEGG: 0 (FDR)

IF LG~CG-motif THEN G-597236-0.6
G-597236-0.6: 0.01185 (562,65,53,12)
Cov-neg: G-180540 G-198157 G-254402 G-258714 G-258873 G-271570 G-282545 G-417685 G-419541 G-547880 G-558970 G-560588 G-566261 G-567196 G-572432 G-574284 G-575185 G-580510 G-592022 G-646180 G-647353 G-652073 G-654168 G-656287 G-669100 G-670882 G-671385 G-677905 G-705737 G-706048 G-710398 G-715463 G-717022 G-722365 G-737207 G-798453 G-801999 G-802304 G-806997 G-813989 G-816631 G-818688 G-821016 G-822136 G-822298 G-824960 G-825940 G-826261 G-827624 G-827935 G-828679 G-832349 TF-287849
Cov-pos: G-260731 G-572020 G-597236 G-662200 G-712554 G-715100 G-743149 G-744851 G-818614 G-820261 G-832630 TF-639804
Significance threshold GO: 0 (FDR)
```

Significance threshold KEGG: 0 (FDR)

IF BN~TATA-box AND NT~as-2-box AND ST~AT1-motif AND ST~Unnamed__1 THEN G-814965-0.55
G-814965-0.55: 0.018427 (562,27,34,5)
Cov-neg: G-198984 G-218480 G-264335 G-548349 G-580490 G-592237 G-593861 G-645963 G-647016 G-653119 G-664200 G-706048 G-714171 G-718004 G-719214 G-725763 G-726819 G-738571 G-748355 G-835598 TF-710397 TF-827145
Cov-pos: G-179914 G-241370 G-572955 G-802304 G-814965
Significance threshold GO: 0.0123348182692343 (FDR)
GO:0022613: P: ribonucleoprotein complex biogenesis: 0.000492347462377412 (537,5,21,3) (88) Genes: G-179914 G-572955 G-802304
GO:0042254: P: ribosome biogenesis: 0.000492347462377412 (537,5,21,3) (88) Genes: G-179914 G-572955 G-802304
GO:0019843: F: rRNA binding: 0.000742800418804488 (537,5,24,3) (88) Genes: G-179914 G-572955 G-592237 G-802304
GO:0003735: F: structural constituent of ribosome: 0.00274548754731334 (537,5,37,3) (88) Genes: G-179914 G-572955 G-592237 G-802304
GO:0006412: P: translation: 0.00399696735833331 (537,5,42,3) (88) Genes: G-179914 G-572955 G-592237 G-802304
GO:0044267: P: cellular protein metabolic process: 0.00401122967924823 (537,5,95,4) (88) Genes: G-179914 G-218480 G-572955 G-592237 G-647016 G-802304 G-814965
GO:0015934: C: large ribosomal subunit: 0.00441672733418986 (537,5,12,2) (88) Genes: G-572955 G-802304
GO:0019538: P: protein metabolic process: 0.0054932826119247 (537,5,103,4) (88) Genes: G-179914 G-218480 G-572955 G-592237 G-647016 G-802304 G-814965
GO:0005840: C: ribosome: 0.00556226784864258 (537,5,47,3) (88) Genes: G-179914 G-572955 G-592237 G-647016 G-725763 G-802304
GO:0003723: F: RNA binding: 0.00705870245888177 (537,5,51,3) (88) Genes: G-179914 G-572955 G-592237 G-802304
GO:0030529: C: ribonucleoprotein complex: 0.00925547458204301 (537,5,56,3) (88) Genes: G-179914 G-572955 G-592237 G-647016 G-725763 G-802304
GO:0044434: C: chloroplast part: 0.0107265704236678 (537,5,218,5) (88) Genes: G-179914 G-198984 G-241370 G-572955 G-592237 G-645963 G-647016 G-664200 G-725763 G-726819 G-738571 G-802304 G-814965
GO:0044435: C: plastid part: 0.0117572895531182 (537,5,222,5) (88) Genes: G-179914 G-198984 G-241370 G-572955 G-592237 G-645963 G-647016 G-664200 G-725763 G-726819 G-738571 G-802304 G-814965
GO:0033279: C: ribosomal subunit: 0.0123348182692343 (537,5,20,2) (88) Genes: G-572955 G-802304
Significance threshold KEGG: 0 (FDR)

IF AS~TATA-box AND PS~ATCT-motif AND ZM~TATA-box THEN G-834497-0.55
G-834497-0.55: 0.000497 (562,25,17,5)
Cov-neg: G-253476 G-254402 G-267832 G-274041 G-281479 G-282545 G-290157 G-571855 G-589333 G-645978 G-653119 G-706393 G-718770 G-724963 G-725575 G-727763 G-798453 G-814158 G-829783 G-831063
Cov-pos: G-571263 G-731248 G-744667 G-834497 TF-643213
Significance threshold GO: 0 (FDR)
Significance threshold KEGG: 0 (FDR)

IF LG~rbcS-CMA7a THEN G-709829-0.55
G-709829-0.55: 0.000796 (562,54,29,9)
Cov-neg: G-172100 G-198157 G-206841 G-242343 G-245181 G-250348 G-254678 G-293436 G-410455 G-411030 G-569972 G-571793 G-572020 G-576166 G-580161 G-582552 G-592573 G-640334 G-643350 G-646230 G-647933 G-649116 G-654065 G-656287 G-671385 G-709740 G-710398 G-715775 G-721810 G-731676 G-734314 G-736262 G-746197 G-799516 G-814158 G-816631 G-824944 G-826111 G-826261 G-832238 G-833338 G-836585 TF-663774 TF-710397 TF-740041
Cov-pos: G-229824 G-266518 G-549690 G-589333 G-680311 G-709829 G-718770 G-745105 G-815682
Significance threshold GO: 0.00776163206437245 (FDR)
GO:0003735: F: structural constituent of ribosome: 0.000122413548214497 (537,9,37,5) (103) Genes: G-229824 G-266518 G-549690 G-709829 G-718770
GO:0006412: P: translation: 0.000231133202484514 (537,9,42,5) (103) Genes: G-229824 G-266518 G-549690 G-709829 G-718770
GO:0005840: C: ribosome: 0.000403143562560958 (537,9,47,5) (103) Genes: G-198157 G-229824 G-266518 G-549690 G-709829 G-718770
GO:0030529: C: ribonucleoprotein complex: 0.000944949208161767 (537,9,56,5) (103) Genes: G-198157 G-229824 G-245181 G-266518 G-549690 G-709829 G-718770
GO:0009507: C: chloroplast: 0.00533704216525947 (537,9,302,9) (103) Genes: G-198157 G-206841 G-229824 G-242343 G-245181 G-250348 G-254678 G-266518 G-293436 G-410455 G-411030 G-549690 G-569972 G-571793 G-580161 G-582552 G-589333 G-640334 G-643350 G-647933 G-649116 G-654065 G-680311 G-709740 G-709829 G-710398 G-715775 G-718770 G-721810 G-731676 G-745105 G-799516 G-814158 G-815682 G-816631 G-826111 G-832238
GO:0009536: C: plastid: 0.00566823046150617 (537,9,304,9) (103) Genes: G-198157 G-206841 G-229824 G-242343 G-245181 G-250348 G-254678 G-266518 G-293436 G-410455 G-411030 G-549690 G-569972 G-571793 G-580161 G-582552 G-589333 G-640334 G-643350 G-647933 G-649116 G-654065 G-680311 G-709740 G-709829 G-710398 G-715775 G-718770 G-721810 G-731676 G-745105 G-799516 G-814158 G-815682 G-816631 G-826111 G-832238
GO:0009059: P: macromolecule biosynthetic process: 0.00737283392002107 (537,9,87,5) (103) Genes: G-229824 G-266518 G-549690 G-576166 G-709829 G-718770 G-833338 TF-710397
GO:0034645: P: cellular macromolecule biosynthetic process: 0.00737283392002107 (537,9,87,5) (103) Genes: G-229824 G-266518 G-549690 G-576166 G-709829 G-718770 G-833338 TF-710397
GO:0010467: P: gene expression: 0.00776163206437245 (537,9,88,5) (103) Genes: G-229824 G-266518 G-549690 G-709829 G-718770 TF-710397
Significance threshold KEGG: 0.038848920863319 (FDR)

KEGG: Ribosome: 0.038848920863319 (140,2,28,2) (1) Genes: G-229824 G-266518

IF GM~GA-motif AND ST~Unnamed__1 THEN G-577759-0.55
G-577759-0.55: 0.014683 (562,65,48,11)
Cov-neg: G-202265 G-250546 G-253476 G-254402 G-257508 G-266518 G-281479 G-282545 G-296649 G-411030 G-414772 G-421706 G-421814 G-549292 G-552356 G-559187 G-560859 G-568456 G-571159 G-571297 G-571855 G-572618 G-576659 G-580490 G-642244 G-654168 G-662200 G-674736 G-705701 G-717926 G-725596 G-725763 G-726819 G-756703 G-784099 G-806997 G-814158 G-814533 G-815682 G-822940 G-825921 G-825945 G-828679 G-831805 G-832349 G-832984 G-835242 G-835648 G-837468 TF-218677 TF-643871 TF-715838 TF-827145 TF-836808
Cov-pos: G-261089 G-577759 G-578419 G-589310 G-657316 G-664200 G-735683 G-738571 G-741765 G-818640 G-822298
Significance threshold GO: 0.0100005849826008 (FDR)
GO:0006091: P: generation of precursor metabolites and energy: 0.000848520650114259 (537,11,67,6) (218) Genes: G-250546 G-261089 G-414772 G-568456 G-589310 G-664200 G-674736 G-705701 G-735683 G-741765 G-818640
GO:0034357: C: photosynthetic membrane: 0.00115552243677648 (537,11,136,8) (218) Genes: G-250546 G-261089 G-266518 G-296649 G-414772 G-421706 G-568456 G-589310 G-654168 G-657316 G-664200 G-674736 G-725763 G-738571 G-741765 G-815682 G-818640 G-822298 G-835648
GO:0019684: P: photosynthesis, light reaction: 0.00116477940139314 (537,11,46,5) (218) Genes: G-250546 G-414772 G-568456 G-589310 G-664200 G-674736 G-735683 G-741765 G-818640
GO:0009521: C: photosystem: 0.00189863054963914 (537,11,51,5) (218) Genes: G-568456 G-589310 G-664200 G-674736 G-741765 G-818640 G-822298
GO:0015979: P: photosynthesis: 0.00316142641984321 (537,11,85,6) (218) Genes: G-250546 G-414772 G-568456 G-589310 G-664200 G-674736 G-735683 G-741765 G-818640 G-822298
GO:0009526: C: plastid envelope: 0.00408964124029182 (537,11,123,7) (218) Genes: G-250546 G-261089 G-414772 G-559187 G-568456 G-589310 G-657316 G-664200 G-725596 G-725763 G-726819 G-735683 G-738571 G-818640 G-835648 TF-218677
GO:0009579: C: thylakoid: 0.00465387818907827 (537,11,165,8) (218) Genes: G-250546 G-261089 G-266518 G-296649 G-414772 G-421706 G-568456 G-571297 G-589310 G-654168 G-657316 G-664200 G-674736 G-705701 G-725763 G-738571 G-741765 G-815682 G-818640 G-822298 G-835648
GO:0031967: C: organelle envelope: 0.00498102976530035 (537,11,127,7) (218) Genes: G-250546 G-261089 G-414772 G-559187 G-568456 G-576659 G-589310 G-657316 G-664200 G-725596 G-725763 G-726819 G-735683 G-738571 G-818640 G-835648 TF-218677
GO:0031975: C: envelope: 0.00498102976530035 (537,11,127,7) (218) Genes: G-250546 G-261089 G-414772 G-559187 G-568456 G-576659 G-589310 G-657316 G-664200 G-725596 G-725763 G-726819 G-735683 G-738571 G-818640 G-835648 TF-218677
GO:0009535: C: chloroplast thylakoid membrane: 0.0069090824829002 (537,11,134,7) (218) Genes: G-250546 G-261089 G-266518 G-296649 G-414772 G-421706 G-568456 G-589310 G-654168 G-657316 G-664200 G-674736 G-725763 G-738571 G-815682 G-818640 G-822298 G-835648
GO:0055035: C: plastid thylakoid membrane: 0.0069090824829002 (537,11,134,7) (218) Genes: G-250546 G-261089 G-266518 G-296649 G-414772 G-421706 G-568456 G-589310 G-654168 G-657316 G-664200 G-674736 G-725763 G-738571 G-815682 G-818640 G-822298 G-835648
GO:0042651: C: thylakoid membrane: 0.00722706735935305 (537,11,135,7) (218) Genes: G-250546 G-261089 G-266518 G-296649 G-414772 G-421706 G-568456 G-589310 G-654168 G-657316 G-664200 G-674736 G-725763 G-738571 G-815682 G-818640 G-822298 G-835648
GO:0044436: C: thylakoid part: 0.00789776262952454 (537,11,137,7) (218) Genes: G-250546 G-261089 G-266518 G-296649 G-414772 G-421706 G-568456 G-589310 G-654168 G-657316 G-664200 G-674736 G-725763 G-738571 G-815682 G-818640 G-822298 G-835648
GO:0009534: C: chloroplast thylakoid: 0.00825105454439856 (537,11,138,7) (218) Genes: G-250546 G-261089 G-266518 G-296649 G-414772 G-421706 G-568456 G-589310 G-654168 G-657316 G-664200 G-674736 G-725763 G-738571 G-815682 G-818640 G-822298 G-835648
GO:0031976: C: plastid thylakoid: 0.00825105454439856 (537,11,138,7) (218) Genes: G-250546 G-261089 G-266518 G-296649 G-414772 G-421706 G-568456 G-589310 G-654168 G-657316 G-664200 G-674736 G-725763 G-738571 G-815682 G-818640 G-822298 G-835648
GO:0031984: C: organelle subcompartment: 0.00825105454439856 (537,11,138,7) (218) Genes: G-250546 G-261089 G-266518 G-296649 G-414772 G-421706 G-568456 G-589310 G-654168 G-657316 G-664200 G-674736 G-725763 G-738571 G-815682 G-818640 G-822298 G-835648
GO:0043234: C: protein complex: 0.00831369553875884 (537,11,102,6) (218) Genes: G-261089 G-568456 G-572618 G-576659 G-589310 G-642244 G-664200 G-674736 G-741765 G-756703 G-818640 G-822298 G-831805
GO:0009768: P: photosynthesis, light harvesting in photosystem I: 0.0100005849826008 (537,11,8,2) (218) Genes: G-589310 G-741765
Significance threshold KEGG: 0 (FDR)

IF GM~Unnamed__6 AND M00343-P$RAV1_01 AND ST~Unnamed__1 THEN G-654168-0.55
G-654168-0.55: 5.742517e-06 (562,19,10,5)
Cov-neg: G-250546 G-293436 G-572354 G-572618 G-577759 G-642244 G-672749 G-725763 G-729481 G-740033 G-760146 G-829854 G-835035 TF-710397
Cov-pos: G-281479 G-589559 G-654168 G-664872 G-830495
Significance threshold GO: 0 (FDR)
Significance threshold KEGG: 0.0123329907502553 (FDR)
KEGG: Photosynthesis - antenna proteins: 0.0123329907502553 (140,2,16,2) (1) Genes: G-664872 G-830495

IF NT~TATA-box THEN G-823432-0.55
G-823432-0.55: 0.000985 (562,54,42,11)
Cov-neg: G-172100 G-207685 G-219367 G-227384 G-242343 G-251580 G-267832 G-282545 G-409286 G-414772 G-415807 G-549292 G-564524 G-564881 G-566261 G-570826 G-571855 G-574284 G-580161 G-591443 G-645978 G-646516 G-672749 G-706048 G-706715 G-714062 G-715100 G-717040 G-729481 G-737939 G-780725 G-815682 G-817211 G-822136 G-822298 G-824944 G-826745 G-827055 G-829783 G-835035 G-835574 TF-643213 TF-715838

Cov-pos: G-254603 G-266518 G-279999 G-571297 G-592237 G-647016 G-760260 G-816669 G-819086 G-822660 G-823432
Significance threshold GO: 0.0128633442333987 (FDR)
GO:0009570: C: chloroplast stroma: 0.000289990172964762 (537,11,113,8) (227) Genes: G-227384 G-266518 G-267832 G-414772 G-571297 G-592237 G-647016 G-706715 G-760260 G-816669 G-817211 G-819086 G-822298 G-823432 G-827055
GO:0010555: P: response to mannitol stimulus: 0.000382167375413594 (537,11,2,2) (227) Genes: G-647016 G-816669
GO:0010275: P: NAD(P)H dehydrogenase complex assembly: 0.000382167375413594 (537,11,2,2) (227) Genes: G-647016 G-816669
GO:0009532: C: plastid stroma: 0.000456160725590908 (537,11,120,8) (227) Genes: G-227384 G-266518 G-267832 G-414772 G-571297 G-592237 G-647016 G-706715 G-760260 G-816669 G-817211 G-819086 G-822298 G-823432 G-827055
GO:0044267: P: cellular protein metabolic process: 0.000793103397622984 (537,11,95,7) (227) Genes: G-254603 G-266518 G-282545 G-571297 G-592237 G-647016 G-737939 G-760260 G-780725 G-816669
GO:0000097: P: sulfur amino acid biosynthetic process: 0.00113364415846912 (537,11,3,2) (227) Genes: G-647016 G-816669
GO:0006534: P: cysteine metabolic process: 0.00113364415846912 (537,11,3,2) (227) Genes: G-647016 G-816669
GO:0019344: P: cysteine biosynthetic process: 0.00113364415846912 (537,11,3,2) (227) Genes: G-647016 G-816669
GO:0005840: C: ribosome: 0.00129049571319308 (537,11,47,5) (227) Genes: G-254603 G-266518 G-282545 G-592237 G-647016 G-737939 G-816669
GO:0019538: P: protein metabolic process: 0.00133683458318372 (537,11,103,7) (227) Genes: G-254603 G-266518 G-267832 G-282545 G-571297 G-592237 G-647016 G-737939 G-760260 G-780725 G-816669
GO:0009507: C: chloroplast: 0.00164099239366111 (537,11,302,11) (227) Genes: G-207685 G-227384 G-242343 G-254603 G-266518 G-267832 G-279999 G-282545 G-414772 G-415807 G-564524 G-566261 G-570826 G-571297 G-580161 G-592237 G-645978 G-646516 G-647016 G-706715 G-715100 G-737939 G-760260 G-815682 G-816669 G-817211 G-819086 G-822298 G-822660 G-823432 G-827055
GO:0009536: C: plastid: 0.0017667386432856 (537,11,304,11) (227) Genes: G-207685 G-227384 G-242343 G-254603 G-266518 G-267832 G-279999 G-282545 G-414772 G-415807 G-564524 G-566261 G-570826 G-571297 G-580161 G-592237 G-645978 G-646516 G-647016 G-706715 G-715100 G-737939 G-760260 G-815682 G-816669 G-817211 G-819086 G-822298 G-822660 G-823432 G-827055
GO:0006457: P: protein folding: 0.00206569071812345 (537,11,14,3) (227) Genes: G-571297 G-647016 G-816669
GO:0009070: P: serine family amino acid biosynthetic process: 0.00224186132449328 (537,11,4,2) (227) Genes: G-647016 G-816669
GO:0009069: P: serine family amino acid metabolic process: 0.00224186132449328 (537,11,4,2) (227) Genes: G-647016 G-816669
GO:0016859: F: cis-trans isomerase activity: 0.00224186132449328 (537,11,4,2) (227) Genes: G-647016 G-816669
GO:0042277: F: peptide binding: 0.00224186132449328 (537,11,4,2) (227) Genes: G-647016 G-816669 G-835035
GO:0003755: F: peptidyl-prolyl cis-trans isomerase activity: 0.00224186132449328 (537,11,4,2) (227) Genes: G-647016 G-816669
GO:0030529: C: ribonucleoprotein complex: 0.00293563340572379 (537,11,56,5) (227) Genes: G-254603 G-266518 G-282545 G-592237 G-647016 G-717040 G-737939 G-816669
GO:0044249: P: cellular biosynthetic process: 0.00342197427961679 (537,11,158,8) (227) Genes: G-254603 G-266518 G-279999 G-282545 G-592237 G-647016 G-706048 G-737939 G-760260 G-816669 G-823432 G-824944 G-829783 G-835574 TF-643213 TF-715838
GO:0006605: P: protein targeting: 0.00372926141402096 (537,11,17,3) (227) Genes: G-571297 G-647016 G-816669
GO:0044271: P: cellular nitrogen compound biosynthetic process: 0.00402243228122216 (537,11,36,4) (227) Genes: G-279999 G-647016 G-760260 G-816669
GO:0009058: P: biosynthetic process: 0.00408748071208917 (537,11,162,8) (227) Genes: G-254603 G-266518 G-279999 G-282545 G-592237 G-647016 G-706048 G-737939 G-760260 G-816669 G-823432 G-824944 G-829783 G-835574 TF-643213 TF-715838
GO:0000096: P: sulfur amino acid metabolic process: 0.0054796551355524 (537,11,6,2) (227) Genes: G-647016 G-816669
GO:0009533: C: chloroplast stromal thylakoid: 0.0054796551355524 (537,11,6,2) (227) Genes: G-647016 G-816669
GO:0006886: P: intracellular protein transport: 0.00696815159331759 (537,11,21,3) (227) Genes: G-571297 G-647016 G-706048 G-816669
GO:0045036: P: protein targeting to chloroplast: 0.00758549326557102 (537,11,7,2) (227) Genes: G-647016 G-816669
GO:0034613: P: cellular protein localization: 0.00797657096087374 (537,11,22,3) (227) Genes: G-571297 G-647016 G-706048 G-816669
GO:0009543: C: chloroplast thylakoid lumen: 0.00906860102162914 (537,11,23,3) (227) Genes: G-647016 G-816669 G-819086 G-822298
GO:0031978: C: plastid thylakoid lumen: 0.00906860102162914 (537,11,23,3) (227) Genes: G-647016 G-816669 G-819086 G-822298
GO:0070727: P: cellular macromolecule localization: 0.00906860102162914 (537,11,23,3) (227) Genes: G-571297 G-647016 G-706048 G-816669
GO:0015031: P: protein transport: 0.0102460186468761 (537,11,24,3) (227) Genes: G-571297 G-647016 G-706048 G-816669
GO:0019843: F: rRNA binding: 0.0102460186468761 (537,11,24,3) (227) Genes: G-254603 G-266518 G-282545 G-592237
GO:0045184: P: establishment of protein localization: 0.0102460186468761 (537,11,24,3) (227) Genes: G-571297 G-647016 G-706048 G-816669
GO:0016053: P: organic acid biosynthetic process: 0.0102460186468761 (537,11,24,3) (227) Genes: G-647016 G-816669 G-823432 G-824944
GO:0046394: P: carboxylic acid biosynthetic process: 0.0102460186468761 (537,11,24,3) (227) Genes: G-647016 G-816669 G-823432 G-824944
GO:0044272: P: sulfur compound biosynthetic process: 0.0127137314970128 (537,11,9,2) (227) Genes: G-647016 G-816669
GO:0008652: P: cellular amino acid biosynthetic process: 0.0127137314970128 (537,11,9,2) (227) Genes: G-647016 G-816669
GO:0046907: P: intracellular transport: 0.0128633442333987 (537,11,26,3) (227) Genes: G-571297 G-647016 G-706048 G-816669
Significance threshold KEGG: 0.0252704976609014 (FDR)
KEGG: Ribosome: 0.0252704976609014 (140,4,28,3) (2) Genes: G-254603 G-266518 G-282545 G-592237

IF GM~Unnamed__2 AND GM~Unnamed__7 THEN G-817211-0.55
G-817211-0.55: 0.004662 (562,53,37,9)

Cov-neg: G-172390 G-198157 G-204684 G-204897 G-207685 G-240319 G-250348 G-267999 G-271570 G-415807 G-421706 G-559187 G-564642 G-564881 G-567196 G-571855 G-572618 G-576481 G-582552 G-595585 G-642574 G-652073 G-652151 G-662808 G-680311 G-715463 G-720677 G-721765 G-723969 G-724072 G-725114 G-726819 G-731296 G-784623 G-799516 G-819059 G-819086 G-821685 G-825331 G-825720 G-826253 G-828771 G-833033 TF-209269
Cov-pos: G-173755 G-230966 G-555320 G-712554 G-791055 G-816237 G-817211 G-831715 G-836765
Significance threshold GO: 0.0104938843488605 (FDR)
GO:0008937: F: ferredoxin reductase activity: 0.000743891880855952 (537,9,3,2) (287) Genes: G-173755 G-817211
GO:0016731: F: oxidoreductase activity, acting on iron-sulfur proteins as donors, NAD or NADP as acceptor: 0.000743891880855952 (537,9,3,2) (287) Genes: G-173755 G-817211
GO:0009535: C: chloroplast thylakoid membrane: 0.00120225246209403 (537,9,134,7) (287) Genes: G-198157 G-230966 G-240319 G-415807 G-421706 G-555320 G-576481 G-642574 G-652073 G-652151 G-680311 G-712554 G-715463 G-731296 G-799516 G-816237 G-817211 G-819086 G-831715 G-836765
GO:0055035: C: plastid thylakoid membrane: 0.00120225246209403 (537,9,134,7) (287) Genes: G-198157 G-230966 G-240319 G-415807 G-421706 G-555320 G-576481 G-642574 G-652073 G-652151 G-680311 G-712554 G-715463 G-731296 G-799516 G-816237 G-817211 G-819086 G-831715 G-836765
GO:0042651: C: thylakoid membrane: 0.00126273175107923 (537,9,135,7) (287) Genes: G-198157 G-230966 G-240319 G-415807 G-421706 G-555320 G-576481 G-642574 G-652073 G-652151 G-680311 G-712554 G-715463 G-731296 G-799516 G-816237 G-817211 G-819086 G-831715 G-836765
GO:0034357: C: photosynthetic membrane: 0.00132570914328743 (537,9,136,7) (287) Genes: G-198157 G-230966 G-240319 G-415807 G-421706 G-555320 G-576481 G-642574 G-652073 G-652151 G-680311 G-712554 G-715463 G-731296 G-799516 G-816237 G-817211 G-819086 G-831715 G-836765
GO:0044436: C: thylakoid part: 0.00139126458565884 (537,9,137,7) (287) Genes: G-198157 G-230966 G-240319 G-415807 G-421706 G-555320 G-576481 G-642574 G-652073 G-652151 G-680311 G-712554 G-715463 G-731296 G-799516 G-816237 G-817211 G-819086 G-831715 G-833033 G-836765
GO:0009534: C: chloroplast thylakoid: 0.00145947977746691 (537,9,138,7) (287) Genes: G-198157 G-230966 G-240319 G-415807 G-421706 G-555320 G-576481 G-642574 G-652073 G-652151 G-680311 G-712554 G-715463 G-731296 G-799516 G-816237 G-817211 G-819086 G-831715 G-836765
GO:0031976: C: plastid thylakoid: 0.00145947977746691 (537,9,138,7) (287) Genes: G-198157 G-230966 G-240319 G-415807 G-421706 G-555320 G-576481 G-642574 G-652073 G-652151 G-680311 G-712554 G-715463 G-731296 G-799516 G-816237 G-817211 G-819086 G-831715 G-836765
GO:0031984: C: organelle subcompartment: 0.00145947977746691 (537,9,138,7) (287) Genes: G-198157 G-230966 G-240319 G-415807 G-421706 G-555320 G-576481 G-642574 G-652073 G-652151 G-680311 G-712554 G-715463 G-731296 G-799516 G-816237 G-817211 G-819086 G-831715 G-836765
GO:0016730: F: oxidoreductase activity, acting on iron-sulfur proteins as donors: 0.00147480233643577 (537,9,4,2) (287) Genes: G-173755 G-817211
GO:0010319: C: stromule: 0.00322773270241729 (537,9,20,3) (287) Genes: G-712554 G-816237 G-831715
GO:0055114: P: oxidation reduction: 0.00352183916106357 (537,9,74,5) (287) Genes: G-173755 G-198157 G-555320 G-652151 G-680311 G-731296 G-816237 G-817211 G-831715
GO:0009579: C: thylakoid: 0.00465573483743957 (537,9,165,7) (287) Genes: G-198157 G-230966 G-240319 G-415807 G-421706 G-555320 G-576481 G-642574 G-652073 G-652151 G-680311 G-712554 G-715463 G-731296 G-799516 G-816237 G-817211 G-819086 G-831715 G-833033 G-836765
GO:0034404: P: nucleobase, nucleoside and nucleotide biosynthetic process: 0.00846912761354285 (537,9,9,2) (287) Genes: G-555320 G-712554
GO:0034654: P: nucleobase, nucleoside, nucleotide and nucleic acid biosynthetic process: 0.00846912761354285 (537,9,9,2) (287) Genes: G-555320 G-712554
GO:0022900: P: electron transport chain: 0.00865717504767915 (537,9,28,3) (287) Genes: G-555320 G-652151 G-680311 G-731296 G-816237 G-817211
GO:0048046: C: apoplast: 0.0102448256239541 (537,9,58,4) (287) Genes: G-198157 G-250348 G-571855 G-712554 G-816237 G-817211 G-831715 G-833033
GO:0015977: P: carbon fixation: 0.0104938843488605 (537,9,10,2) (287) Genes: G-198157 G-712554 G-831715
GO:0019685: P: photosynthesis, dark reaction: 0.0104938843488605 (537,9,10,2) (287) Genes: G-198157 G-712554 G-831715
GO:0071704: P: organic substance metabolic process: 0.0104938843488605 (537,9,10,2) (287) Genes: G-198157 G-712554 G-831715
GO:0019253: P: reductive pentose-phosphate cycle: 0.0104938843488605 (537,9,10,2) (287) Genes: G-198157 G-712554 G-831715
Significance threshold KEGG: 0 (FDR)

IF AT~TATA-box AND LE~circadian AND ST~Unnamed__1 THEN G-197763-0.6
G-197763-0.6: 0.001038 (562,35,21,6)
Cov-neg: G-253476 G-276236 G-298673 G-414772 G-550847 G-568456 G-571297 G-572020 G-572354 G-575716 G-589289 G-592237 G-642244 G-646516 G-653119 G-706048 G-718004 G-737939 G-750752 G-780257 G-784099 G-784623 G-818614 G-818640 G-820932 G-835035 G-835598 TF-232345 TF-710397
Cov-pos: G-197763 G-245181 G-725763 G-731296 G-802304 G-826253
Significance threshold GO: 0 (FDR)
Significance threshold KEGG: 0 (FDR)

IF AT~ATC-motif AND AT~NON-box THEN G-640334-0.5
G-640334-0.5: 0.000059 (562,43,21,8)
Cov-neg: G-206001 G-217802 G-253476 G-254603 G-416054 G-566261 G-566934 G-570873 G-571263 G-593861 G-595045 G-644646 G-647353 G-649116 G-654077 G-655455 G-656287 G-664221 G-668020 G-711610 G-715100 G-732807 G-734314 G-736262 G-745105 G-747465 G-761926 G-817206 G-821685 G-822177 G-824462 G-827624 G-831371 TF-232345 TF-640573
Cov-pos: G-572020 G-580510 G-640334 G-710398 G-712554 G-715775 G-734671 G-831715
Significance threshold GO: 0 (FDR)
Significance threshold KEGG: 0.0431266538657268 (FDR)
KEGG: Carbon fixation in photosynthetic organisms: 0.0431266538657268 (140,4,13,2) (3) Genes: G-712554 G-831715

IF AS~TATA-box AND BO~ELI-box3 THEN G-826191-0.5
G-826191-0.5: 0.001795 (562,33,33,7)
Cov-neg: G-206841 G-230630 G-264335 G-274035 G-421706 G-549486 G-558898 G-571159 G-571263 G-576525 G-589333 G-642574 G-645629 G-645963 G-657316 G-667705 G-673777 G-675419 G-717040 G-743910 G-814694 G-817545 G-818359 G-825854 G-829783 TF-643871
Cov-pos: G-230966 G-565812 G-664200 G-714062 G-815393 G-820821 G-826191
Significance threshold GO: 0.00410761449723011 (FDR)
GO:0009534: C: chloroplast thylakoid: 0.00146146077419749 (537,7,138,6) (101) Genes: G-206841 G-230966 G-421706 G-549486 G-565812 G-589333 G-642574 G-645963 G-657316 G-664200 G-743910 G-815393 G-820821 G-826191
GO:0031976: C: plastid thylakoid: 0.00146146077419749 (537,7,138,6) (101) Genes: G-206841 G-230966 G-421706 G-549486 G-565812 G-589333 G-642574 G-645963 G-657316 G-664200 G-743910 G-815393 G-820821 G-826191
GO:0031984: C: organelle subcompartment: 0.00146146077419749 (537,7,138,6) (101) Genes: G-206841 G-230966 G-421706 G-549486 G-565812 G-589333 G-642574 G-645963 G-657316 G-664200 G-743910 G-815393 G-820821 G-826191
GO:0022900: P: electron transport chain: 0.00387319041371268 (537,7,28,3) (101) Genes: G-565812 G-645629 G-645963 G-664200 G-814694 G-826191
GO:0009579: C: thylakoid: 0.00410761449723011 (537,7,165,6) (101) Genes: G-206841 G-230966 G-421706 G-549486 G-565812 G-576525 G-589333 G-642574 G-645963 G-657316 G-664200 G-743910 G-815393 G-820821 G-826191
Significance threshold KEGG: 0 (FDR)

IF NT~E2Fa THEN TF-639804-0.5
TF-639804-0.5: 0.002126 (562,49,43,10)
Cov-neg: G-247314 G-249644 G-298709 G-417459 G-547880 G-550607 G-551453 G-557551 G-567316 G-572432 G-572618 G-573011 G-575185 G-587540 G-640334 G-652073 G-652151 G-655445 G-662808 G-680311 G-706754 G-712747 G-715463 G-722365 G-724072 G-725114 G-725127 G-749916 G-752731 G-784197 G-809857 G-820612 G-821599 G-826191 G-835910 TF-287849 TF-420425 TF-643213 TF-819435
Cov-pos: G-227384 G-416054 G-581162 G-595585 G-711610 G-712554 G-734671 G-735683 G-816237 TF-639804
Significance threshold GO: 0.000256605362061963 (FDR)
GO:0010319: C: stromule: 0.000256605362061963 (537,10,20,4) (399) Genes: G-227384 G-572432 G-712554 G-712747 G-735683 G-816237
Significance threshold KEGG: 0.0431266538657268 (FDR)
KEGG: Carbon fixation in photosynthetic organisms: 0.0431266538657268 (140,4,13,2) (6) Genes: G-567316 G-712554 G-735683

IF AT~AE-box AND GM~Unnamed__6 AND M00479-P$Alfin1_Q2 THEN G-833331-0.5
G-833331-0.5: 0.000771 (562,21,22,5)
Cov-neg: G-180540 G-250348 G-267999 G-551203 G-559187 G-567196 G-642306 G-655136 G-706048 G-724072 G-736262 G-814376 G-819059 G-828771 G-833033 TF-286321
Cov-pos: G-576481 G-642244 G-722365 G-822660 G-833331
Significance threshold GO: 0 (FDR)
Significance threshold KEGG: 0 (FDR)

IF GM~Unnamed__6 AND TA~G-Box AND ZM~Unnamed__14 THEN G-198157-0.5
G-198157-0.5: 0.000104 (562,21,15,5)
Cov-neg: G-271570 G-419541 G-571754 G-571855 G-664872 G-722365 G-723969 G-740496 G-740771 G-743910 G-819059 G-822660 G-824944 G-827935 G-830089 G-831371
Cov-pos: G-198157 G-551203 G-580797 G-652073 G-715463
Significance threshold GO: 0.0254661249440245 (FDR)
GO:0048492: C: ribulose bisphosphate carboxylase complex: 6.94849773479325e-05 (537,5,2,2) (118) Genes: G-198157 G-580797
GO:0009853: P: photorespiration: 6.94849773479325e-05 (537,5,2,2) (118) Genes: G-198157 G-580797
GO:0009573: C: chloroplast ribulose bisphosphate carboxylase complex: 6.94849773479325e-05 (537,5,2,2) (118) Genes: G-198157 G-580797
GO:0016984: F: ribulose-bisphosphate carboxylase activity: 6.94849773479325e-05 (537,5,2,2) (118) Genes: G-198157 G-580797
GO:0015979: P: photosynthesis: 8.98077574496272e-05 (537,5,85,5) (118) Genes: G-198157 G-551203 G-571754 G-580797 G-652073 G-664872 G-715463 G-743910 G-830089
GO:0043234: C: protein complex: 0.000228045492436513 (537,5,102,5) (118) Genes: G-198157 G-551203 G-580797 G-652073 G-664872 G-715463 G-743910 G-819059 G-827935 G-830089
GO:0016831: F: carboxy-lyase activity: 0.00041379715891693 (537,5,4,2) (118) Genes: G-198157 G-580797
GO:0009769: P: photosynthesis, light harvesting in photosystem II: 0.00041379715891693 (537,5,4,2) (118) Genes: G-652073 G-715463
GO:0009941: C: chloroplast envelope: 0.000567536885121064 (537,5,122,5) (118) Genes: G-198157 G-271570 G-551203 G-571754 G-580797 G-652073 G-664872 G-715463 G-819059
GO:0009526: C: plastid envelope: 0.000591585058219657 (537,5,123,5) (118) Genes: G-198157 G-271570 G-551203 G-571754 G-580797 G-652073 G-664872 G-715463 G-819059
GO:0043094: P: cellular metabolic compound salvage: 0.000687078944415705 (537,5,5,2) (118) Genes: G-198157 G-580797
GO:0031967: C: organelle envelope: 0.000696064928496575 (537,5,127,5) (118) Genes: G-198157 G-271570 G-551203 G-571754 G-580797 G-652073 G-664872 G-715463 G-819059

GO:0031975: C: envelope: 0.000696064928496575 (537,5,127,5) (118) Genes: G-198157 G-271570 G-551203 G-571754 G-580797 G-652073 G-664872 G-715463 G-819059
GO:0046872: F: metal ion binding: 0.000783711058438603 (537,5,130,5) (118) Genes: G-198157 G-551203 G-580797 G-652073 G-715463 G-743910
GO:0009535: C: chloroplast thylakoid membrane: 0.000914078923033855 (537,5,134,5) (118) Genes: G-198157 G-551203 G-571754 G-580797 G-652073 G-664872 G-715463 G-743910 G-827935 G-830089
GO:0055035: C: plastid thylakoid membrane: 0.000914078923033855 (537,5,134,5) (118) Genes: G-198157 G-551203 G-571754 G-580797 G-652073 G-664872 G-715463 G-743910 G-827935 G-830089
GO:0042651: C: thylakoid membrane: 0.000949235804688813 (537,5,135,5) (118) Genes: G-198157 G-551203 G-571754 G-580797 G-652073 G-664872 G-715463 G-743910 G-827935 G-830089
GO:0043169: F: cation binding: 0.000949235804688813 (537,5,135,5) (118) Genes: G-198157 G-551203 G-580797 G-652073 G-715463 G-743910
GO:0034357: C: photosynthetic membrane: 0.000985466178914232 (537,5,136,5) (118) Genes: G-198157 G-551203 G-571754 G-580797 G-652073 G-664872 G-715463 G-743910 G-827935 G-830089
GO:0043167: F: ion binding: 0.00102279444326757 (537,5,137,5) (118) Genes: G-198157 G-551203 G-580797 G-652073 G-715463 G-743910
GO:0044436: C: thylakoid part: 0.00102279444326757 (537,5,137,5) (118) Genes: G-198157 G-551203 G-571754 G-580797 G-652073 G-664872 G-715463 G-743910 G-827935 G-830089
GO:0009534: C: chloroplast thylakoid: 0.00106124536218697 (537,5,138,5) (118) Genes: G-198157 G-551203 G-571754 G-580797 G-652073 G-664872 G-715463 G-743910 G-827935 G-830089
GO:0031976: C: plastid thylakoid: 0.00106124536218697 (537,5,138,5) (118) Genes: G-198157 G-551203 G-571754 G-580797 G-652073 G-664872 G-715463 G-743910 G-827935 G-830089
GO:0031984: C: organelle subcompartment: 0.00106124536218697 (537,5,138,5) (118) Genes: G-198157 G-551203 G-571754 G-580797 G-652073 G-664872 G-715463 G-743910 G-827935 G-830089
GO:0031977: C: thylakoid lumen: 0.0013216984372719 (537,5,29,3) (118) Genes: G-198157 G-551203 G-580797
GO:0016830: F: carbon-carbon lyase activity: 0.00143206304463141 (537,5,7,2) (118) Genes: G-198157 G-580797
GO:0009523: C: photosystem II: 0.00232626830688805 (537,5,35,3) (118) Genes: G-551203 G-652073 G-715463 G-743910 G-830089
GO:0009579: C: thylakoid: 0.00262479242343216 (537,5,165,5) (118) Genes: G-198157 G-551203 G-571754 G-580797 G-652073 G-664872 G-715463 G-743910 G-827935 G-830089
GO:0015977: P: carbon fixation: 0.00303422809235365 (537,5,10,2) (118) Genes: G-198157 G-580797
GO:0019685: P: photosynthesis, dark reaction: 0.00303422809235365 (537,5,10,2) (118) Genes: G-198157 G-580797
GO:0071704: P: organic substance metabolic process: 0.00303422809235365 (537,5,10,2) (118) Genes: G-198157 G-580797
GO:0019253: P: reductive pentose-phosphate cycle: 0.00303422809235365 (537,5,10,2) (118) Genes: G-198157 G-580797
GO:0018298: P: protein-chromophore linkage: 0.00520007291386284 (537,5,13,2) (118) Genes: G-652073 G-715463 G-743910
GO:0004497: F: monooxygenase activity: 0.00520007291386284 (537,5,13,2) (118) Genes: G-198157 G-580797
GO:0019684: P: photosynthesis, light reaction: 0.00522277141545503 (537,5,46,3) (118) Genes: G-551203 G-652073 G-664872 G-715463 G-743910
GO:0016829: F: lyase activity: 0.00604383875532744 (537,5,14,2) (118) Genes: G-198157 G-580797
GO:0016051: P: carbohydrate biosynthetic process: 0.00694729740010277 (537,5,15,2) (118) Genes: G-198157 G-580797 G-831371
GO:0009521: C: photosystem: 0.00705870245888177 (537,5,51,3) (118) Genes: G-551203 G-652073 G-715463 G-743910 G-830089
GO:0010218: P: response to far red light: 0.0100086192014787 (537,5,18,2) (118) Genes: G-198157 G-580797 G-664872 G-743910
GO:0048046: C: apoplast: 0.010240439624002 (537,5,58,3) (118) Genes: G-198157 G-551203 G-571855 G-580797
GO:0044434: C: chloroplast part: 0.0107265704236678 (537,5,218,5) (118) Genes: G-198157 G-271570 G-551203 G-571754 G-580797 G-652073 G-664872 G-715463 G-743910 G-819059 G-827935 G-830089
GO:0022626: C: cytosolic ribosome: 0.0111436592406034 (537,5,19,2) (118) Genes: G-198157 G-580797
GO:0009637: P: response to blue light: 0.0111436592406034 (537,5,19,2) (118) Genes: G-198157 G-580797 G-664872 G-743910
GO:0044435: C: plastid part: 0.0117572895531182 (537,5,222,5) (118) Genes: G-198157 G-271570 G-551203 G-571754 G-580797 G-652073 G-664872 G-715463 G-743910 G-819059 G-827935 G-830089
GO:0030076: C: light-harvesting complex: 0.0123348182692343 (537,5,20,2) (118) Genes: G-652073 G-664872 G-715463 G-743910
GO:0010114: P: response to red light: 0.0135813941436373 (537,5,21,2) (118) Genes: G-198157 G-580797 G-664872 G-743910
GO:0009765: P: photosynthesis, light harvesting: 0.0148826889009027 (537,5,22,2) (118) Genes: G-652073 G-664872 G-715463 G-743910
GO:0006091: P: generation of precursor metabolites and energy: 0.0154685416280635 (537,5,67,3) (118) Genes: G-551203 G-652073 G-664872 G-715463 G-743910
GO:0016168: F: chlorophyll binding: 0.016238008747964 (537,5,23,2) (118) Genes: G-652073 G-664872 G-715463 G-743910
GO:0009639: P: response to red or far red light: 0.0176466640505886 (537,5,24,2) (118) Genes: G-198157 G-580797 G-664872 G-743910
GO:0005507: F: copper ion binding: 0.0206212432147072 (537,5,26,2) (118) Genes: G-198157 G-580797
GO:0042170: C: plastid membrane: 0.0254661249440245 (537,5,29,2) (118) Genes: G-198157 G-571754 G-580797 G-819059
GO:0046906: F: tetrapyrrole binding: 0.0254661249440245 (537,5,29,2) (118) Genes: G-652073 G-664872 G-715463 G-743910
GO:0031969: C: chloroplast membrane: 0.0254661249440245 (537,5,29,2) (118) Genes: G-198157 G-571754 G-580797 G-819059
Significance threshold KEGG: 0.0030385629384693 (FDR)
KEGG: Glyoxylate and dicarboxylate metabolism: 0.0030385629384693 (140,5,3,2) (4) Genes: G-198157 G-580797

IF M00182-P$GBP_Q6 AND ZM~TATA-box AND ZM~Unnamed__14 THEN G-822688-0.6

G-822688-0.6: 0.000046 (562,41,16,7)
Cov-neg: G-198157 G-260250 G-267832 G-279999 G-410455 G-414772 G-421814 G-552732 G-566261 G-570340 G-571855 G-573011 G-589559 G-592408 G-653119 G-670620 G-706284 G-706393 G-715522 G-716206 G-732598 G-741765 G-750752 G-784099 G-814376 G-815682 G-820612 G-823432 G-824944 G-829854 G-835242 TF-218677 TF-643200 TF-740041
Cov-pos: G-568456 G-569972 G-664872 G-743910 G-745105 G-821685 G-822688
Significance threshold GO: 0.00215518066060858 (FDR)
GO:0030076: C: light-harvesting complex: 0.00141143306469434 (537,7,20,3) (136) Genes: G-568456 G-664872 G-743910
GO:0015979: P: photosynthesis: 0.00144342861035593 (537,7,85,5) (136) Genes: G-198157 G-414772 G-566261 G-568456 G-569972 G-664872 G-716206 G-732598 G-741765 G-743910 G-745105 G-829854
GO:0009765: P: photosynthesis, light harvesting: 0.00188488744623907 (537,7,22,3) (136) Genes: G-568456 G-664872 G-741765 G-743910
GO:0016168: F: chlorophyll binding: 0.00215518066060858 (537,7,23,3) (136) Genes: G-568456 G-664872 G-743910 G-815682
Significance threshold KEGG: 0.00464851857010162 (FDR)
KEGG: Photosynthesis - antenna proteins: 0.00464851857010162 (140,4,16,3) (2) Genes: G-568456 G-664872 G-743910

IF HV~GAG-motif AND M00735-P$ZAP1_01 THEN G-820850-0.6
G-820850-0.6: 0.014992 (562,25,35,5)
Cov-neg: G-174965 G-269072 G-293436 G-409286 G-550607 G-557890 G-558970 G-569931 G-587540 G-649116 G-664934 G-668020 G-706754 G-720677 G-725596 G-736262 G-746197 TF-268609 TF-556378 TF-827145
Cov-pos: G-417685 G-709002 G-737207 G-761926 G-820850
Significance threshold GO: 0.0117572895531182 (FDR)
GO:0016021: C: integral to membrane: 0.000897550794220633 (537,5,65,4) (137) Genes: G-409286 G-417685 G-664934 G-668020 G-709002 G-737207 G-820850
GO:0009535: C: chloroplast thylakoid membrane: 0.000914078923033855 (537,5,134,5) (137) Genes: G-417685 G-649116 G-664934 G-668020 G-706754 G-709002 G-737207 G-761926 G-820850
GO:0055035: C: plastid thylakoid membrane: 0.000914078923033855 (537,5,134,5) (137) Genes: G-417685 G-649116 G-664934 G-668020 G-706754 G-709002 G-737207 G-761926 G-820850
GO:0042651: C: thylakoid membrane: 0.000949235804688813 (537,5,135,5) (137) Genes: G-417685 G-649116 G-664934 G-668020 G-706754 G-709002 G-737207 G-761926 G-820850
GO:0034357: C: photosynthetic membrane: 0.000985466178914232 (537,5,136,5) (137) Genes: G-417685 G-649116 G-664934 G-668020 G-706754 G-709002 G-737207 G-761926 G-820850
GO:0044436: C: thylakoid part: 0.00102279444326757 (537,5,137,5) (137) Genes: G-417685 G-649116 G-664934 G-668020 G-706754 G-709002 G-737207 G-761926 G-820850
GO:0009534: C: chloroplast thylakoid: 0.00106124536218697 (537,5,138,5) (137) Genes: G-417685 G-649116 G-664934 G-668020 G-706754 G-709002 G-737207 G-761926 G-820850
GO:0031976: C: plastid thylakoid: 0.00106124536218697 (537,5,138,5) (137) Genes: G-417685 G-649116 G-664934 G-668020 G-706754 G-709002 G-737207 G-761926 G-820850
GO:0031984: C: organelle subcompartment: 0.00106124536218697 (537,5,138,5) (137) Genes: G-417685 G-649116 G-664934 G-668020 G-706754 G-709002 G-737207 G-761926 G-820850
GO:0031224: C: intrinsic to membrane: 0.00175846494599759 (537,5,77,4) (137) Genes: G-409286 G-417685 G-569931 G-664934 G-668020 G-709002 G-737207 G-820850
GO:0015979: P: photosynthesis: 0.00259555506406766 (537,5,85,4) (137) Genes: G-664934 G-668020 G-709002 G-737207 G-761926 G-820850
GO:0009579: C: thylakoid: 0.00262479242343216 (537,5,165,5) (137) Genes: G-417685 G-649116 G-664934 G-668020 G-706754 G-709002 G-737207 G-761926 G-820850
GO:0043234: C: protein complex: 0.00528925922763692 (537,5,102,4) (137) Genes: G-417685 G-557890 G-664934 G-668020 G-709002 G-737207 G-820850
GO:0044425: C: membrane part: 0.00659980082928132 (537,5,108,4) (137) Genes: G-409286 G-417685 G-569931 G-664934 G-668020 G-709002 G-737207 G-820850
GO:0009521: C: photosystem: 0.00705870245888177 (537,5,51,3) (137) Genes: G-664934 G-668020 G-709002 G-737207 G-820850
GO:0044434: C: chloroplast part: 0.0107265704236678 (537,5,218,5) (137) Genes: G-293436 G-417685 G-550607 G-649116 G-664934 G-668020 G-706754 G-709002 G-725596 G-737207 G-761926 G-820850
GO:0044435: C: plastid part: 0.0117572895531182 (537,5,222,5) (137) Genes: G-293436 G-417685 G-550607 G-649116 G-664934 G-668020 G-706754 G-709002 G-725596 G-737207 G-761926 G-820850
Significance threshold KEGG: 0.0340139005447986 (FDR)
KEGG: Photosynthesis: 0.0340139005447986 (140,4,31,3) (3) Genes: G-417685 G-709002 G-820850

IF OS~TGGCA THEN G-820232-0.55
G-820232-0.55: 0.00149 (562,24,22,5)
Cov-neg: G-267999 G-424279 G-564827 G-567196 G-569931 G-569972 G-659520 G-721810 G-813989 G-817727 G-820261 G-825921 G-825945 G-827624 G-830089 G-831371 TF-286321 TF-556378 TF-594467
Cov-pos: G-217802 G-580510 G-712554 G-814694 G-820232
Significance threshold GO: 0 (FDR)
Significance threshold KEGG: 0.022771348138875 (FDR)

KEGG: Carbon fixation in photosynthetic organisms: 0.022771348138875 (140,3,13,2) (3) Genes: G-712554 G-721810 G-820232

IF HV~CE1 AND ZM~Unnamed__17 THEN G-668020-0.5
G-668020-0.5: 0.000125 (562,20,37,7)
Cov-neg: G-179024 G-249644 G-254603 G-282545 G-288961 G-582742 G-595585 G-680311 G-822177 G-823815 G-829854 G-832984 TF-830919
Cov-pos: G-590521 G-652073 G-668020 G-715100 G-744851 G-817544 G-827624
Significance threshold GO: 0.0322113656615413 (FDR)
GO:0015979: P: photosynthesis: 1.35049259322698e-05 (537,6,85,6) (127) Genes: G-652073 G-668020 G-715100 G-744851 G-817544 G-827624 G-829854
GO:0009521: C: photosystem: 3.58086092640189e-05 (537,6,51,5) (127) Genes: G-652073 G-668020 G-715100 G-744851 G-817544 G-829854
GO:0016021: C: integral to membrane: 0.000122936469667136 (537,6,65,5) (127) Genes: G-179024 G-652073 G-668020 G-715100 G-744851 G-817544
GO:0009522: C: photosystem I: 0.000126813942300479 (537,6,31,4) (127) Genes: G-652073 G-668020 G-744851 G-817544 G-829854
GO:0009523: C: photosystem II: 0.000208397846953397 (537,6,35,4) (127) Genes: G-652073 G-668020 G-715100 G-817544
GO:0018298: P: protein-chromophore linkage: 0.000213607113930826 (537,6,13,3) (127) Genes: G-652073 G-668020 G-817544
GO:0009535: C: chloroplast thylakoid membrane: 0.000221646956900858 (537,6,134,6) (127) Genes: G-652073 G-668020 G-680311 G-715100 G-744851 G-817544 G-827624
GO:0055035: C: plastid thylakoid membrane: 0.000221646956900858 (537,6,134,6) (127) Genes: G-652073 G-668020 G-680311 G-715100 G-744851 G-817544 G-827624
GO:0042651: C: thylakoid membrane: 0.000231956117687219 (537,6,135,6) (127) Genes: G-652073 G-668020 G-680311 G-715100 G-744851 G-817544 G-827624 G-829854
GO:0034357: C: photosynthetic membrane: 0.000242661784657356 (537,6,136,6) (127) Genes: G-652073 G-668020 G-680311 G-715100 G-744851 G-817544 G-827624 G-829854
GO:0044436: C: thylakoid part: 0.000253776064870657 (537,6,137,6) (127) Genes: G-652073 G-668020 G-680311 G-715100 G-744851 G-817544 G-827624 G-829854
GO:0009534: C: chloroplast thylakoid: 0.000265311340546893 (537,6,138,6) (127) Genes: G-652073 G-668020 G-680311 G-715100 G-744851 G-817544 G-827624
GO:0031976: C: plastid thylakoid: 0.000265311340546893 (537,6,138,6) (127) Genes: G-652073 G-668020 G-680311 G-715100 G-744851 G-817544 G-827624
GO:0031984: C: organelle subcompartment: 0.000265311340546893 (537,6,138,6) (127) Genes: G-652073 G-668020 G-680311 G-715100 G-744851 G-817544 G-827624
GO:0031224: C: intrinsic to membrane: 0.000287965496132819 (537,6,77,5) (127) Genes: G-179024 G-652073 G-668020 G-715100 G-744851 G-817544
GO:0019684: P: photosynthesis, light reaction: 0.000627231977766835 (537,6,46,4) (127) Genes: G-652073 G-668020 G-744851 G-817544
GO:0009579: C: thylakoid: 0.000789411255168131 (537,6,165,6) (127) Genes: G-652073 G-668020 G-680311 G-715100 G-744851 G-817544 G-827624 G-829854
GO:0030076: C: light-harvesting complex: 0.000826251714450876 (537,6,20,3) (127) Genes: G-652073 G-668020 G-817544
GO:0009765: P: photosynthesis, light harvesting: 0.00110657598188966 (537,6,22,3) (127) Genes: G-652073 G-668020 G-817544
GO:0043234: C: protein complex: 0.00116037433839499 (537,6,102,5) (127) Genes: G-652073 G-668020 G-715100 G-744851 G-817544 G-829854
GO:0016168: F: chlorophyll binding: 0.0012670743848292 (537,6,23,3) (127) Genes: G-652073 G-668020 G-817544
GO:0044425: C: membrane part: 0.00153571792081311 (537,6,108,5) (127) Genes: G-179024 G-652073 G-668020 G-715100 G-744851 G-817544 G-829854
GO:0046906: F: tetrapyrrole binding: 0.00254707346675217 (537,6,29,3) (127) Genes: G-652073 G-668020 G-817544
GO:0006091: P: generation of precursor metabolites and energy: 0.00275180453182562 (537,6,67,4) (127) Genes: G-652073 G-668020 G-680311 G-744851 G-817544
GO:0009941: C: chloroplast envelope: 0.00278114409682374 (537,6,122,5) (127) Genes: G-179024 G-652073 G-668020 G-744851 G-817544 G-822177 G-827624
GO:0009526: C: plastid envelope: 0.00289342917572836 (537,6,123,5) (127) Genes: G-179024 G-652073 G-668020 G-744851 G-817544 G-822177 G-827624
GO:0031967: C: organelle envelope: 0.00337827001011073 (537,6,127,5) (127) Genes: G-179024 G-652073 G-668020 G-744851 G-817544 G-822177 G-827624
GO:0031975: C: envelope: 0.00337827001011073 (537,6,127,5) (127) Genes: G-179024 G-652073 G-668020 G-744851 G-817544 G-822177 G-827624
GO:0043687: P: post-translational protein modification: 0.00341246120581316 (537,6,32,3) (127) Genes: G-652073 G-668020 G-817544
GO:0044434: C: chloroplast part: 0.00429466071473831 (537,6,218,6) (127) Genes: G-179024 G-652073 G-668020 G-680311 G-715100 G-744851 G-817544 G-822177 G-827624
GO:0044435: C: plastid part: 0.00479573652824384 (537,6,222,6) (127) Genes: G-179024 G-652073 G-668020 G-680311 G-715100 G-744851 G-817544 G-822177 G-827624
GO:0006464: P: protein modification process: 0.00482690492383488 (537,6,36,3) (127) Genes: G-652073 G-668020 G-817544
GO:0010287: C: plastoglobule: 0.0065625706288182 (537,6,40,3) (127) Genes: G-652073 G-668020 G-817544
GO:0043412: P: macromolecule modification: 0.0065625706288182 (537,6,40,3) (127) Genes: G-652073 G-668020 G-817544
GO:0016020: C: membrane: 0.0143534410116876 (537,6,266,6) (127) Genes: G-179024 G-652073 G-668020 G-680311 G-715100 G-744851 G-817544 G-822177 G-827624 G-829854 G-832984
GO:0010218: P: response to far red light: 0.0147146616193729 (537,6,18,2) (127) Genes: G-668020 G-817544
GO:0009637: P: response to blue light: 0.0163628165634251 (537,6,19,2) (127) Genes: G-668020 G-817544
GO:0010114: P: response to red light: 0.0198921845078219 (537,6,21,2) (127) Genes: G-668020 G-817544
GO:0009639: P: response to red or far red light: 0.0257490798891663 (537,6,24,2) (127) Genes: G-668020 G-817544 G-822177
GO:0051186: P: cofactor metabolic process: 0.0278462726996315 (537,6,25,2) (127) Genes: G-744851 G-827624
GO:0044446: C: intracellular organelle part: 0.0279766057532186 (537,6,297,6) (127) Genes: G-179024 G-595585 G-652073 G-668020 G-680311 G-715100 G-744851 G-817544 G-822177 G-827624

GO:0009507: C: chloroplast: 0.0309503553200292 (537,6,302,6) (127) Genes: G-179024 G-254603 G-282545 G-652073 G-668020 G-680311 G-715100 G-744851 G-817544 G-822177 G-827624 G-829854 TF-830919
GO:0009536: C: plastid: 0.0322113656615413 (537,6,304,6) (127) Genes: G-179024 G-254603 G-282545 G-652073 G-668020 G-680311 G-715100 G-744851 G-817544 G-822177 G-827624 G-829854 TF-830919
Significance threshold KEGG: 0.0107937172206739 (FDR)
KEGG: Photosynthesis - antenna proteins: 0.0107937172206739 (140,5,16,3) (2) Genes: G-652073 G-668020 G-817544

IF AS~TATA-box AND AT~TATA-box AND BN~TATA-box AND PC~Box_4 AND ZM~TATA-box THEN G-823432-0.55
G-823432-0.55: 0.003659 (562,72,42,12)
Cov-neg: G-206841 G-241370 G-253476 G-274041 G-282545 G-293436 G-552356 G-552732 G-558898 G-564827 G-565812 G-570826 G-571373 G-571855 G-585475 G-589289 G-595585 G-642574 G-646230 G-647353 G-670620 G-674736 G-675419 G-706048 G-706284 G-716206 G-717040 G-719214 G-725575 G-734314 G-741765 G-743910 G-744667 G-750752 G-778626 G-814158 G-814965 G-817451 G-821630 G-822434 G-822940 G-825854 G-828416 G-829783 G-830112 G-830598 G-831805 G-832984 G-834497 G-834944 G-835242 G-835598 G-836444 TF-232345 TF-576309 TF-643200 TF-643871 TF-834586 TF-835874 TF-836808
Cov-pos: G-254402 G-571297 G-572955 G-576525 G-592237 G-647016 G-670039 G-705737 G-730367 G-740033 G-820932 G-823432
Significance threshold GO: 0.00463300525126385 (FDR)
GO:0009570: C: chloroplast stroma: 5.48628450580846e-06 (537,12,113,10) (330) Genes: G-241370 G-254402 G-293436 G-571297 G-572955 G-576525 G-592237 G-647016 G-670039 G-705737 G-716206 G-730367 G-743910 G-821630 G-823432 G-830112
GO:0009532: C: plastid stroma: 9.95742656970114e-06 (537,12,120,10) (330) Genes: G-241370 G-254402 G-293436 G-571297 G-572955 G-576525 G-592237 G-647016 G-670039 G-705737 G-716206 G-730367 G-743910 G-821630 G-823432 G-830112
GO:0044434: C: chloroplast part: 1.6660579083691e-05 (537,12,218,12) (330) Genes: G-206841 G-241370 G-254402 G-274041 G-293436 G-564827 G-565812 G-571297 G-572955 G-576525 G-592237 G-642574 G-647016 G-670039 G-674736 G-705737 G-716206 G-730367 G-740033 G-743910 G-744667 G-814965 G-820932 G-821630 G-823432 G-830112 G-834944
GO:0044435: C: plastid part: 2.08402476241191e-05 (537,12,222,12) (330) Genes: G-206841 G-241370 G-254402 G-274041 G-293436 G-564827 G-565812 G-571297 G-572955 G-576525 G-592237 G-642574 G-647016 G-670039 G-674736 G-705737 G-716206 G-730367 G-740033 G-743910 G-744667 G-814965 G-820932 G-821630 G-823432 G-830112 G-834944
GO:0044267: P: cellular protein metabolic process: 0.000196572492245186 (537,12,95,8) (330) Genes: G-254402 G-282545 G-571297 G-572955 G-576525 G-592237 G-647016 G-670039 G-705737 G-743910 G-814965
GO:0019538: P: protein metabolic process: 0.000360983580435465 (537,12,103,8) (330) Genes: G-254402 G-282545 G-571297 G-572955 G-576525 G-592237 G-647016 G-670039 G-705737 G-743910 G-814965 G-822940
GO:0044446: C: intracellular organelle part: 0.000740232119091901 (537,12,297,12) (330) Genes: G-206841 G-241370 G-254402 G-274041 G-293436 G-564827 G-565812 G-571297 G-572955 G-576525 G-589289 G-592237 G-595585 G-642574 G-647016 G-670039 G-670620 G-674736 G-705737 G-706048 G-716206 G-717040 G-730367 G-740033 G-743910 G-744667 G-814965 G-820932 G-821630 G-823432 G-825854 G-828416 G-830112 G-831805 G-834944 G-835242 G-836444 TF-232345
GO:0009507: C: chloroplast: 0.000907849404098513 (537,12,302,12) (330) Genes: G-206841 G-241370 G-254402 G-274041 G-282545 G-293436 G-564827 G-565812 G-570826 G-571297 G-572955 G-576525 G-592237 G-642574 G-647016 G-670039 G-670620 G-674736 G-705737 G-706284 G-716206 G-730367 G-740033 G-741765 G-743910 G-744667 G-750752 G-814158 G-814965 G-820932 G-821630 G-822940 G-823432 G-830112 G-834944 G-835598 TF-576309 TF-834586
GO:0009536: C: plastid: 0.000984133883047083 (537,12,304,12) (330) Genes: G-206841 G-241370 G-254402 G-274041 G-282545 G-293436 G-564827 G-565812 G-570826 G-571297 G-572955 G-576525 G-592237 G-642574 G-647016 G-670039 G-670620 G-674736 G-705737 G-706284 G-716206 G-730367 G-740033 G-741765 G-743910 G-744667 G-750752 G-814158 G-814965 G-820932 G-821630 G-822940 G-823432 G-830112 G-834944 G-835598 TF-576309 TF-834586
GO:0019843: F: rRNA binding: 0.00120273498521296 (537,12,24,4) (330) Genes: G-282545 G-572955 G-592237 G-670039 G-705737
GO:0006412: P: translation: 0.0012148448430254 (537,12,42,5) (330) Genes: G-254402 G-282545 G-572955 G-592237 G-670039 G-705737
GO:0005840: C: ribosome: 0.00206719171015231 (537,12,47,5) (330) Genes: G-282545 G-572955 G-592237 G-647016 G-670039 G-705737 G-830112
GO:0030529: C: ribonucleoprotein complex: 0.00463300525126385 (537,12,56,5) (330) Genes: G-282545 G-572955 G-592237 G-647016 G-670039 G-705737 G-717040 G-830112 G-831805 G-835242
Significance threshold KEGG: 0.00731936190178236 (FDR)
KEGG: Ribosome: 0.00731936190178236 (140,3,28,3) (1) Genes: G-282545 G-572955 G-592237 G-705737

IF AT~GAG-motif AND AT~OCT AND M00479-P$Alfin1_Q2 THEN G-712747-0.55
G-712747-0.55: 0.00093 (562,24,20,5)
Cov-neg: G-180540 G-197108 G-207685 G-217802 G-250348 G-260250 G-260731 G-271570 G-293436 G-416054 G-548349 G-652073 G-667705 G-715463 G-756336 G-822298 G-833204 G-836585 TF-556378
Cov-pos: G-712747 G-722365 G-739211 G-806997 G-822177
Significance threshold GO: 0.0124001086872471 (FDR)
GO:0006457: P: protein folding: 0.000137477865076948 (537,5,14,3) (141) Genes: G-712747 G-739211 G-822177
GO:0009409: P: response to cold: 0.00321024727682169 (537,5,39,3) (141) Genes: G-667705 G-712747 G-739211 G-822177
GO:0044267: P: cellular protein metabolic process: 0.00401122967924823 (537,5,95,4) (141) Genes: G-180540 G-217802 G-652073 G-667705 G-712747 G-715463 G-739211 G-756336 G-806997 G-822177 G-833204
GO:0009266: P: response to temperature stimulus: 0.0048967075343084 (537,5,45,3) (141) Genes: G-667705 G-712747 G-739211 G-822177
GO:0019538: P: protein metabolic process: 0.0054932826119247 (537,5,103,4) (141) Genes: G-180540 G-217802 G-652073 G-667705 G-712747 G-715463 G-739211 G-756336 G-806997 G-822177 G-833204

GO:0005524: F: ATP binding: 0.00705870245888177 (537,5,51,3) (141) Genes: G-217802 G-712747 G-739211 G-822177 G-836585
GO:0009570: C: chloroplast stroma: 0.00785888393910268 (537,5,113,4) (141) Genes: G-180540 G-197108 G-293436 G-712747 G-739211 G-806997 G-822177 G-822298 G-833204
GO:0032559: F: adenyl ribonucleotide binding: 0.00789277420116459 (537,5,53,3) (141) Genes: G-217802 G-712747 G-739211 G-822177 G-836585
GO:0030554: F: adenyl nucleotide binding: 0.00925547458204301 (537,5,56,3) (141) Genes: G-217802 G-712747 G-739211 G-822177 G-836585
GO:0001882: F: nucleoside binding: 0.00925547458204301 (537,5,56,3) (141) Genes: G-217802 G-712747 G-739211 G-822177 G-836585
GO:0001883: F: purine nucleoside binding: 0.00925547458204301 (537,5,56,3) (141) Genes: G-217802 G-712747 G-739211 G-822177 G-836585
GO:0009532: C: plastid stroma: 0.00990044682602916 (537,5,120,4) (141) Genes: G-180540 G-197108 G-293436 G-712747 G-739211 G-806997 G-822177 G-822298 G-833204
GO:0032553: F: ribonucleotide binding: 0.0107563913339705 (537,5,59,3) (141) Genes: G-180540 G-217802 G-712747 G-739211 G-822177 G-836585
GO:0032555: F: purine ribonucleotide binding: 0.0107563913339705 (537,5,59,3) (141) Genes: G-180540 G-217802 G-712747 G-739211 G-822177 G-836585
GO:0017076: F: purine nucleotide binding: 0.0124001086872471 (537,5,62,3) (141) Genes: G-180540 G-217802 G-712747 G-739211 G-822177 G-836585
GO:0005739: C: mitochondrion: 0.0124001086872471 (537,5,62,3) (141) Genes: G-652073 G-712747 G-715463 G-739211 G-822177
Significance threshold KEGG: 0 (FDR)

IF GM~alpha4 AND ZM~Unnamed__14 AND ZM~Unnamed__4 THEN G-267832-0.5
G-267832-0.5: 0.000028 (562,29,9,5)
Cov-neg: G-204897 G-206001 G-240319 G-271570 G-414772 G-550847 G-551203 G-564524 G-571855 G-572618 G-580797 G-641534 G-642383 G-643350 G-652073 G-712554 G-715463 G-721810 G-723969 G-820389 G-829854 G-830089 G-832238 G-835910
Cov-pos: G-267832 G-267999 G-570340 G-648266 G-817727
Significance threshold GO: 0 (FDR)
Significance threshold KEGG: 0 (FDR)

IF HV~ATC-motif AND M00182-P$GBP_Q6 THEN TF-218677-0.55
TF-218677-0.55: 0.000891 (562,30,16,5)
Cov-neg: G-172390 G-207685 G-249644 G-260250 G-269043 G-290157 G-419541 G-580797 G-593861 G-644646 G-652073 G-652151 G-680311 G-710398 G-712554 G-715522 G-721765 G-725114 G-740033 G-741765 G-743910 G-745105 G-820612 G-829690 TF-640573
Cov-pos: G-414772 G-578419 G-821063 G-831715 TF-218677
Significance threshold GO: 0 (FDR)
Significance threshold KEGG: 0 (FDR)

IF ZM~GC-motif AND ZM~Unnamed__16 THEN G-728017-0.55
G-728017-0.55: 0.001374 (562,26,20,5)
Cov-neg: G-194941 G-206001 G-227384 G-249644 G-271570 G-293436 G-416054 G-570873 G-580161 G-592022 G-593861 G-641534 G-652073 G-655455 G-661329 G-677905 G-710398 G-723969 G-743149 TF-287849 TF-819435
Cov-pos: G-202723 G-254402 G-282545 G-728017 G-766070
Significance threshold GO: 0.0176466640505886 (FDR)
GO:0006412: P: translation: 0.000154024181857894 (537,5,42,4) (62) Genes: G-194941 G-254402 G-282545 G-728017 G-766070
GO:0003723: F: RNA binding: 0.000338955106369858 (537,5,51,4) (62) Genes: G-202723 G-282545 G-728017 G-766070
GO:0003676: F: nucleic acid binding: 0.000696064928496575 (537,5,127,5) (62) Genes: G-202723 G-254402 G-282545 G-580161 G-593861 G-655455 G-728017 G-766070 TF-287849
GO:0003735: F: structural constituent of ribosome: 0.00274548754731334 (537,5,37,3) (62) Genes: G-194941 G-282545 G-728017 G-766070
GO:0009059: P: macromolecule biosynthetic process: 0.00284360540446789 (537,5,87,4) (62) Genes: G-194941 G-254402 G-282545 G-728017 G-766070 TF-287849 TF-819435
GO:0034645: P: cellular macromolecule biosynthetic process: 0.00284360540446789 (537,5,87,4) (62) Genes: G-194941 G-254402 G-282545 G-728017 G-766070 TF-287849 TF-819435
GO:0010467: P: gene expression: 0.00297390757280183 (537,5,88,4) (62) Genes: G-194941 G-254402 G-282545 G-728017 G-766070 TF-287849 TF-819435
GO:0044267: P: cellular protein metabolic process: 0.00401122967924823 (537,5,95,4) (62) Genes: G-194941 G-254402 G-282545 G-652073 G-710398 G-728017 G-766070
GO:0019538: P: protein metabolic process: 0.0054932826119247 (537,5,103,4) (62) Genes: G-194941 G-254402 G-282545 G-652073 G-710398 G-728017 G-766070
GO:0005840: C: ribosome: 0.00556226784864258 (537,5,47,3) (62) Genes: G-194941 G-282545 G-728017 G-766070
GO:0030529: C: ribonucleoprotein complex: 0.00925547458204301 (537,5,56,3) (62) Genes: G-194941 G-282545 G-728017 G-766070
GO:0033279: C: ribosomal subunit: 0.0123348182692343 (537,5,20,2) (62) Genes: G-728017 G-766070
GO:0019843: F: rRNA binding: 0.0176466640505886 (537,5,24,2) (62) Genes: G-282545 G-766070
Significance threshold KEGG: 0.00731936190178236 (FDR)
KEGG: Ribosome: 0.00731936190178236 (140,3,28,3) (1) Genes: G-282545 G-728017 G-766070

IF HV~ABRE AND ST~Unnamed__1 THEN G-564827-0.6
G-564827-0.6: 0.002163 (562,48,30,8)

Cov-neg: G-247314 G-250348 G-254402 G-269043 G-298673 G-414772 G-558898 G-560859 G-565812 G-571754 G-571855 G-572354 G-578767 G-580490 G-589289 G-589333 G-593861 G-644065 G-709712 G-715522 G-717926 G-722193 G-726819 G-731296 G-738571 G-739211 G-740496 G-750752 G-780257 G-798453 G-814533 G-815393 G-817745 G-822688 G-822940 G-829854 G-834944 G-835242 TF-218677 TF-827145
Cov-pos: G-415807 G-564827 G-566261 G-716206 G-724963 G-732598 G-741765 G-745105
Significance threshold GO: 0.0201633204726502 (FDR)
GO:0009521: C: photosystem: 3.91250613248866e-09 (537,8,51,8) (121) Genes: G-415807 G-564827 G-566261 G-709712 G-716206 G-724963 G-732598 G-741765 G-745105 G-829854
GO:0015979: P: photosynthesis: 2.95694679297344e-07 (537,8,85,8) (121) Genes: G-414772 G-415807 G-564827 G-565812 G-566261 G-571754 G-709712 G-716206 G-724963 G-731296 G-732598 G-741765 G-745105 G-829854
GO:0009522: C: photosystem I: 5.86230381204864e-07 (537,8,31,6) (121) Genes: G-415807 G-564827 G-566261 G-709712 G-724963 G-732598 G-741765 G-829854
GO:0043234: C: protein complex: 1.34742947089804e-06 (537,8,102,8) (121) Genes: G-415807 G-564827 G-566261 G-709712 G-716206 G-724963 G-732598 G-741765 G-745105 G-817745 G-829854
GO:0044425: C: membrane part: 2.16297480468931e-06 (537,8,108,8) (121) Genes: G-269043 G-415807 G-564827 G-566261 G-572354 G-589289 G-709712 G-716206 G-724963 G-726819 G-732598 G-741765 G-745105 G-829854 G-834944
GO:0034357: C: photosynthetic membrane: 1.44598590367335e-05 (537,8,136,8) (121) Genes: G-298673 G-414772 G-415807 G-564827 G-565812 G-566261 G-571754 G-589333 G-709712 G-716206 G-724963 G-731296 G-732598 G-738571 G-739211 G-741765 G-745105 G-829854 G-834944
GO:0009538: C: photosystem I reaction center: 2.15140468124681e-05 (537,8,5,3) (121) Genes: G-564827 G-566261 G-732598
GO:0009579: C: thylakoid: 7.0467219387891e-05 (537,8,165,8) (121) Genes: G-298673 G-414772 G-415807 G-564827 G-565812 G-566261 G-571754 G-589333 G-593861 G-709712 G-716206 G-724963 G-731296 G-732598 G-738571 G-739211 G-741765 G-745105 G-815393 G-829854 G-834944
GO:0010287: C: plastoglobule: 8.51886602218619e-05 (537,8,40,5) (121) Genes: G-564827 G-566261 G-589333 G-709712 G-716206 G-724963 G-732598 G-834944
GO:0009535: C: chloroplast thylakoid membrane: 0.000337812549940134 (537,8,134,7) (121) Genes: G-298673 G-414772 G-415807 G-564827 G-565812 G-566261 G-571754 G-589333 G-709712 G-716206 G-724963 G-731296 G-732598 G-738571 G-739211 G-745105 G-834944
GO:0055035: C: plastid thylakoid membrane: 0.000337812549940134 (537,8,134,7) (121) Genes: G-298673 G-414772 G-415807 G-564827 G-565812 G-566261 G-571754 G-589333 G-709712 G-716206 G-724963 G-731296 G-732598 G-738571 G-739211 G-745105 G-834944
GO:0042651: C: thylakoid membrane: 0.000355542416314874 (537,8,135,7) (121) Genes: G-298673 G-414772 G-415807 G-564827 G-565812 G-566261 G-571754 G-589333 G-709712 G-716206 G-724963 G-731296 G-732598 G-738571 G-739211 G-745105 G-829854 G-834944
GO:0044436: C: thylakoid part: 0.000393365074009816 (537,8,137,7) (121) Genes: G-298673 G-414772 G-415807 G-564827 G-565812 G-566261 G-571754 G-589333 G-709712 G-716206 G-724963 G-731296 G-732598 G-738571 G-739211 G-745105 G-815393 G-829854 G-834944
GO:0009534: C: chloroplast thylakoid: 0.000413513503080982 (537,8,138,7) (121) Genes: G-298673 G-414772 G-415807 G-564827 G-565812 G-566261 G-571754 G-589333 G-709712 G-716206 G-724963 G-731296 G-732598 G-738571 G-739211 G-745105 G-815393 G-834944
GO:0031976: C: plastid thylakoid: 0.000413513503080982 (537,8,138,7) (121) Genes: G-298673 G-414772 G-415807 G-564827 G-565812 G-566261 G-571754 G-589333 G-709712 G-716206 G-724963 G-731296 G-732598 G-738571 G-739211 G-745105 G-815393 G-834944
GO:0031984: C: organelle subcompartment: 0.000413513503080982 (537,8,138,7) (121) Genes: G-298673 G-414772 G-415807 G-564827 G-565812 G-566261 G-571754 G-589333 G-709712 G-716206 G-724963 G-731296 G-732598 G-738571 G-739211 G-745105 G-815393 G-834944
GO:0019684: P: photosynthesis, light reaction: 0.00257080404198495 (537,8,46,4) (121) Genes: G-414772 G-565812 G-709712 G-716206 G-724963 G-731296 G-732598 G-741765
GO:0016020: C: membrane: 0.00343446227384204 (537,8,266,8) (121) Genes: G-250348 G-269043 G-298673 G-414772 G-415807 G-564827 G-565812 G-566261 G-571754 G-572354 G-589289 G-589333 G-593861 G-709712 G-716206 G-724963 G-726819 G-731296 G-732598 G-738571 G-739211 G-741765 G-745105 G-750752 G-815393 G-829854 G-834944 TF-218677
GO:0009768: P: photosynthesis, light harvesting in photosystem I: 0.00520749905302193 (537,8,8,2) (121) Genes: G-709712 G-724963 G-741765
GO:0044434: C: chloroplast part: 0.00893871755542651 (537,8,218,7) (121) Genes: G-254402 G-298673 G-414772 G-415807 G-564827 G-565812 G-566261 G-571754 G-589333 G-709712 G-716206 G-724963 G-726819 G-731296 G-732598 G-738571 G-739211 G-745105 G-815393 G-834944 TF-218677
GO:0009507: C: chloroplast: 0.00960304525652353 (537,8,302,8) (121) Genes: G-250348 G-254402 G-298673 G-414772 G-415807 G-564827 G-565812 G-566261 G-571754 G-589333 G-593861 G-709712 G-716206 G-724963 G-726819 G-731296 G-732598 G-738571 G-739211 G-741765 G-745105 G-750752 G-798453 G-815393 G-822940 G-829854 G-834944 TF-218677
GO:0044435: C: plastid part: 0.0100669058431214 (537,8,222,7) (121) Genes: G-254402 G-298673 G-414772 G-415807 G-560859 G-564827 G-565812 G-566261 G-571754 G-589333 G-709712 G-716206 G-724963 G-726819 G-731296 G-732598 G-738571 G-739211 G-745105 G-815393 G-834944 TF-218677
GO:0009536: C: plastid: 0.0101300470072134 (537,8,304,8) (121) Genes: G-250348 G-254402 G-298673 G-414772 G-415807 G-560859 G-564827 G-565812 G-566261 G-571754 G-589333 G-593861 G-709712 G-716206 G-724963 G-726819 G-731296 G-732598 G-738571 G-739211 G-741765 G-745105 G-750752 G-798453 G-815393 G-822940 G-829854 G-834944 TF-218677
GO:0006091: P: generation of precursor metabolites and energy: 0.0105440650540658 (537,8,67,4) (121) Genes: G-414772 G-565812 G-709712 G-716206 G-724963 G-731296 G-732598 G-741765
GO:0009523: C: photosystem II: 0.0113566548290004 (537,8,35,3) (121) Genes: G-709712 G-716206 G-724963 G-745105
GO:0044237: P: cellular metabolic process: 0.0201633204726502 (537,8,331,8) (121) Genes: G-250348 G-254402 G-298673 G-414772 G-415807 G-558898 G-560859 G-564827 G-565812 G-566261 G-571754 G-571855 G-578767 G-589289 G-709712 G-716206 G-724963 G-726819 G-731296 G-732598 G-739211 G-740496 G-741765 G-745105 G-750752 G-798453 G-817745 G-829854 G-834944 TF-218677 TF-827145
Significance threshold KEGG: 0.000407494117835047 (FDR)
KEGG: Photosynthesis: 0.000407494117835047 (140,5,31,5) (1) Genes: G-415807 G-564827 G-566261 G-716206 G-732598

IF AT~AE-box AND M00439-P$C1_Q2 AND PH~ACE THEN G-674736-0.5
G-674736-0.5: 0.000667 (562,25,18,5)
Cov-neg: G-180540 G-204897 G-227384 G-267999 G-555320 G-571057 G-573011 G-580161 G-595045 G-641273 G-642383 G-643350 G-646516 G-706048 G-814376 G-822660 G-828771 G-829854 G-832630 TF-286321
Cov-pos: G-551203 G-649116 G-674736 G-833033 TF-566736
Significance threshold GO: 0 (FDR)
Significance threshold KEGG: 0 (FDR)

IF M00343-P$RAV1_01 AND PH~ACE THEN G-652151-0.5
G-652151-0.5: 0.000505 (562,60,41,12)
Cov-neg: G-172100 G-174634 G-180540 G-197108 G-217802 G-228329 G-249644 G-267999 G-293436 G-410396 G-410455 G-415487 G-547880 G-555320 G-557360 G-567316 G-568456 G-572432 G-572618 G-578767 G-595045 G-640334 G-641534 G-643350 G-654077 G-662808 G-664872 G-672749 G-680311 G-712554 G-715371 G-715463 G-724072 G-743149 G-746197 G-813709 G-820261 G-820389 G-822079 G-822177 G-822688 G-824944 G-826745 G-828771 G-829854 G-836585 TF-286321 TF-830919
Cov-pos: G-227384 G-416054 G-641273 G-652151 G-671325 G-744851 G-791055 G-799516 G-830089 G-831063 G-832074 G-832238
Significance threshold GO: 0.0127413826159455 (FDR)
GO:0009521: C: photosystem: 1.43428098746698e-06 (537,12,51,8) (229) Genes: G-568456 G-641273 G-643350 G-671325 G-715463 G-744851 G-799516 G-813709 G-820261 G-829854 G-830089 G-831063 G-832074 G-832238
GO:0009535: C: chloroplast thylakoid membrane: 1.60145769505678e-06 (537,12,134,11) (229) Genes: G-180540 G-227384 G-416054 G-555320 G-568456 G-572432 G-595045 G-640334 G-641273 G-643350 G-652151 G-654077 G-664872 G-671325 G-680311 G-712554 G-715463 G-744851 G-799516 G-813709 G-820261 G-830089 G-831063 G-832074 G-832238
GO:0055035: C: plastid thylakoid membrane: 1.60145769505678e-06 (537,12,134,11) (229) Genes: G-180540 G-227384 G-416054 G-555320 G-568456 G-572432 G-595045 G-640334 G-641273 G-643350 G-652151 G-654077 G-664872 G-671325 G-680311 G-712554 G-715463 G-744851 G-799516 G-813709 G-820261 G-830089 G-831063 G-832074 G-832238
GO:0042651: C: thylakoid membrane: 1.7396550279117e-06 (537,12,135,11) (229) Genes: G-180540 G-227384 G-416054 G-555320 G-568456 G-572432 G-595045 G-640334 G-641273 G-643350 G-652151 G-654077 G-664872 G-671325 G-680311 G-712554 G-715463 G-744851 G-799516 G-813709 G-820261 G-829854 G-830089 G-831063 G-832074 G-832238
GO:0034357: C: photosynthetic membrane: 1.88853687098119e-06 (537,12,136,11) (229) Genes: G-180540 G-227384 G-416054 G-555320 G-568456 G-572432 G-595045 G-640334 G-641273 G-643350 G-652151 G-654077 G-664872 G-671325 G-680311 G-712554 G-715463 G-744851 G-799516 G-813709 G-820261 G-829854 G-830089 G-831063 G-832074 G-832238
GO:0044436: C: thylakoid part: 2.04883399054349e-06 (537,12,137,11) (229) Genes: G-180540 G-227384 G-416054 G-555320 G-568456 G-572432 G-595045 G-640334 G-641273 G-643350 G-652151 G-654077 G-664872 G-671325 G-680311 G-712554 G-715463 G-744851 G-799516 G-813709 G-820261 G-829854 G-830089 G-831063 G-832074 G-832238
GO:0009534: C: chloroplast thylakoid: 2.22132063319109e-06 (537,12,138,11) (229) Genes: G-180540 G-227384 G-416054 G-555320 G-568456 G-572432 G-595045 G-640334 G-641273 G-643350 G-652151 G-654077 G-664872 G-671325 G-680311 G-712554 G-715463 G-744851 G-799516 G-813709 G-820261 G-830089 G-831063 G-832074 G-832238
GO:0031976: C: plastid thylakoid: 2.22132063319109e-06 (537,12,138,11) (229) Genes: G-180540 G-227384 G-416054 G-555320 G-568456 G-572432 G-595045 G-640334 G-641273 G-643350 G-652151 G-654077 G-664872 G-671325 G-680311 G-712554 G-715463 G-744851 G-799516 G-813709 G-820261 G-830089 G-831063 G-832074 G-832238
GO:0031984: C: organelle subcompartment: 2.22132063319109e-06 (537,12,138,11) (229) Genes: G-180540 G-227384 G-416054 G-555320 G-568456 G-572432 G-595045 G-640334 G-641273 G-643350 G-652151 G-654077 G-664872 G-671325 G-680311 G-712554 G-715463 G-744851 G-799516 G-813709 G-820261 G-830089 G-831063 G-832074 G-832238
GO:0009579: C: thylakoid: 1.59555235132161e-05 (537,12,165,11) (229) Genes: G-180540 G-227384 G-416054 G-555320 G-567316 G-568456 G-572432 G-595045 G-640334 G-641273 G-643350 G-652151 G-654077 G-664872 G-671325 G-680311 G-712554 G-715463 G-744851 G-799516 G-813709 G-820261 G-820389 G-829854 G-830089 G-831063 G-832074 G-832238
GO:0009523: C: photosystem II: 3.47195042723083e-05 (537,12,35,6) (229) Genes: G-568456 G-641273 G-643350 G-671325 G-715463 G-799516 G-813709 G-820261 G-830089 G-831063 G-832074
GO:0015979: P: photosynthesis: 8.41085803708481e-05 (537,12,85,8) (229) Genes: G-555320 G-567316 G-568456 G-572432 G-641273 G-654077 G-664872 G-671325 G-712554 G-715463 G-744851 G-799516 G-813709 G-820261 G-820389 G-829854 G-830089 G-831063 G-832074 G-832238
GO:0044434: C: chloroplast part: 0.000324760563298142 (537,12,218,11) (229) Genes: G-180540 G-197108 G-217802 G-227384 G-267999 G-293436 G-416054 G-555320 G-567316 G-568456 G-572432 G-595045 G-640334 G-641273 G-641534 G-643350 G-652151 G-654077 G-664872 G-671325 G-680311 G-712554 G-715463 G-743149 G-744851 G-799516 G-813709 G-820261 G-820389 G-822177 G-830089 G-831063 G-832074 G-832238
GO:0043234: C: protein complex: 0.00033560131908606 (537,12,102,8) (229) Genes: G-174634 G-267999 G-555320 G-568456 G-572432 G-572618 G-595045 G-641273 G-643350 G-654077 G-664872 G-671325 G-715463 G-744851 G-799516 G-813709 G-820261 G-826745 G-829854 G-830089 G-831063 G-832074 G-832238
GO:0044435: C: plastid part: 0.00039418686382866 (537,12,222,11) (229) Genes: G-180540 G-197108 G-217802 G-227384 G-267999 G-293436 G-416054 G-555320 G-567316 G-568456 G-572432 G-595045 G-640334 G-641273 G-641534 G-643350 G-652151 G-654077 G-664872 G-671325 G-680311 G-712554 G-715463 G-743149 G-744851 G-799516 G-813709 G-820261 G-820389 G-822177 G-830089 G-831063 G-832074 G-832238
GO:0044425: C: membrane part: 0.000513538111163458 (537,12,108,8) (229) Genes: G-410396 G-547880 G-555320 G-568456 G-572432 G-595045 G-641273 G-643350 G-654077 G-671325 G-672749 G-715463 G-744851 G-799516 G-813709 G-820261 G-829854 G-830089 G-831063 G-832074 G-832238

GO:0010608: P: posttranscriptional regulation of gene expression: 0.000667051488647109 (537,12,9,3) (229) Genes: G-652151 G-744851 G-791055
GO:0031978: C: plastid thylakoid lumen: 0.00101470631502022 (537,12,23,4) (229) Genes: G-641273 G-643350 G-652151 G-831063 G-832238
GO:0009543: C: chloroplast thylakoid lumen: 0.00101470631502022 (537,12,23,4) (229) Genes: G-641273 G-643350 G-652151 G-831063 G-832238
GO:0031977: C: thylakoid lumen: 0.00252720979442388 (537,12,29,4) (229) Genes: G-641273 G-643350 G-652151 G-831063 G-832238
GO:0016020: C: membrane: 0.00264059232556333 (537,12,266,11) (229) Genes: G-180540 G-227384 G-267999 G-410396 G-416054 G-547880 G-555320 G-568456 G-572432 G-595045 G-640334 G-641273 G-643350 G-652151 G-654077 G-664872 G-671325 G-672749 G-680311 G-712554 G-715463 G-744851 G-746197 G-799516 G-813709 G-820261 G-822177 G-826745 G-828771 G-829854 G-830089 G-831063 G-832074 G-832238 TF-286321
GO:0044446: C: intracellular organelle part: 0.00819431779385802 (537,12,297,11) (229) Genes: G-180540 G-197108 G-217802 G-227384 G-267999 G-293436 G-416054 G-547880 G-555320 G-567316 G-568456 G-572432 G-572618 G-595045 G-640334 G-641273 G-641534 G-643350 G-652151 G-654077 G-662808 G-664872 G-671325 G-680311 G-712554 G-715371 G-715463 G-743149 G-744851 G-799516 G-813709 G-820261 G-820389 G-822177 G-826745 G-830089 G-831063 G-832074 G-832238
GO:0030093: C: chloroplast photosystem I: 0.00904556772567769 (537,12,7,2) (229) Genes: G-744851 G-832238
GO:0009507: C: chloroplast: 0.00970556527887126 (537,12,302,11) (229) Genes: G-180540 G-197108 G-217802 G-227384 G-267999 G-293436 G-410455 G-416054 G-555320 G-567316 G-568456 G-572432 G-595045 G-640334 G-641273 G-641534 G-643350 G-652151 G-654077 G-664872 G-671325 G-680311 G-712554 G-715463 G-743149 G-744851 G-799516 G-813709 G-820261 G-820389 G-822079 G-822177 G-829854 G-830089 G-831063 G-832074 G-832238 TF-830919
GO:0009536: C: plastid: 0.0103753910059179 (537,12,304,11) (229) Genes: G-180540 G-197108 G-217802 G-227384 G-267999 G-293436 G-410455 G-416054 G-555320 G-567316 G-568456 G-572432 G-595045 G-640334 G-641273 G-641534 G-643350 G-652151 G-654077 G-664872 G-671325 G-680311 G-712554 G-715463 G-743149 G-744851 G-799516 G-813709 G-820261 G-820389 G-822079 G-822177 G-829854 G-830089 G-831063 G-832074 G-832238 TF-830919
GO:0044444: C: cytoplasmic part: 0.0127413826159455 (537,12,375,12) (229) Genes: G-174634 G-180540 G-197108 G-217802 G-227384 G-249644 G-267999 G-293436 G-410455 G-416054 G-547880 G-555320 G-567316 G-568456 G-572432 G-595045 G-640334 G-641273 G-641534 G-643350 G-652151 G-654077 G-664872 G-671325 G-680311 G-712554 G-715463 G-743149 G-744851 G-791055 G-799516 G-813709 G-820261 G-820389 G-822079 G-822177 G-824944 G-826745 G-828771 G-829854 G-830089 G-831063 G-832074 G-832238 G-836585 TF-830919
Significance threshold KEGG: 0.0020525629639099 (FDR)
KEGG: Photosynthesis: 0.0020525629639099 (140,6,31,5) (2) Genes: G-555320 G-643350 G-652151 G-743149 G-744851 G-799516 G-831063 G-832238

IF AT~ATC-motif AND M00408-P$MADSA_Q2 THEN G-706754-0.5
G-706754-0.5: 0.0002 (562,18,31,6)
Cov-neg: G-253476 G-411030 G-416054 G-417685 G-572020 G-640334 G-711610 G-715522 G-725114 G-725127 G-731248 G-827624
Cov-pos: G-254603 G-549486 G-571038 G-580161 G-706754 G-766070
Significance threshold GO: 0.0028320064108892 (FDR)
GO:0000311: C: plastid large ribosomal subunit: 0.000619143763715988 (537,6,4,2) (122) Genes: G-706754 G-766070
GO:0000315: C: organellar large ribosomal subunit: 0.000619143763715988 (537,6,4,2) (122) Genes: G-706754 G-766070
GO:0009547: C: plastid ribosome: 0.00213468613565011 (537,6,7,2) (122) Genes: G-706754 G-766070
GO:0000313: C: organellar ribosome: 0.0028320064108892 (537,6,8,2) (122) Genes: G-706754 G-766070
Significance threshold KEGG: 0.0252704976609014 (FDR)
KEGG: Ribosome: 0.0252704976609014 (140,4,28,3) (2) Genes: G-254603 G-706754 G-766070

IF M00479-P$Alfin1_Q2 AND M00506-P$LIM1_01 AND PA~Unnamed__1 THEN G-567316-0.5
G-567316-0.5: 0.002146 (562,86,47,15)
Cov-neg: G-172100 G-172390 G-197108 G-206001 G-227384 G-249644 G-267832 G-267999 G-274035 G-279999 G-288961 G-410455 G-416904 G-421814 G-551203 G-557360 G-558970 G-560588 G-560859 G-564642 G-566261 G-569931 G-570340 G-572618 G-573011 G-578419 G-587540 G-592022 G-592408 G-593861 G-640334 G-642383 G-643350 G-644646 G-652073 G-652151 G-653119 G-655445 G-659520 G-668020 G-673777 G-677905 G-680311 G-706393 G-710398 G-714062 G-715371 G-715463 G-720677 G-721765 G-721810 G-722365 G-723778 G-723969 G-746197 G-798453 G-814376 G-817206 G-819059 G-819485 G-820612 G-822298 G-822660 G-828771 G-833496 G-835910 G-837320 TF-286321 TF-287849 TF-640573 TF-652803
Cov-pos: G-229824 G-254603 G-261089 G-271570 G-416054 G-567316 G-647933 G-656599 G-712554 G-743149 G-784099 G-817544 G-830112 G-832238 G-835242
Significance threshold GO: 0 (FDR)
Significance threshold KEGG: 0 (FDR)

IF LE~circadian AND PC~ACE THEN G-572020-0.6
G-572020-0.6: 0.000908 (562,41,31,8)
Cov-neg: G-207685 G-228329 G-234689 G-414772 G-421814 G-573011 G-592408 G-641534 G-652073 G-653119 G-664221 G-706393 G-715371 G-715463 G-723969 G-725763 G-731296 G-750752 G-802304 G-817211 G-817745 G-818614 G-819059 G-819178 G-824944 G-830089 G-831371 G-832825 G-833033 G-835035 G-835910 G-836962 TF-287849
Cov-pos: G-550669 G-572020 G-589310 G-643350 G-726168 G-734671 G-821685 G-826955
Significance threshold GO: 0 (FDR)
Significance threshold KEGG: 0 (FDR)

IF CR~MSA-like AND MA0034.1_Gamyb THEN TF-232345-0.55
TF-232345-0.55: 2.084590e-07 (562,12,9,5)

Cov-neg: G-227384 G-557551 G-571057 G-580797 G-641120 G-825854 TF-830919
Cov-pos: G-593861 G-655445 G-835910 TF-232345 TF-571462
Significance threshold GO: 0.0111436592406034 (FDR)
GO:0034728: P: nucleosome organization: 7.21002472134454e-08 (537,5,22,5) (57) Genes: G-571057 G-593861 G-655445 G-835910 TF-232345 TF-571462
GO:0006334: P: nucleosome assembly: 7.21002472134454e-08 (537,5,22,5) (57) Genes: G-571057 G-593861 G-655445 G-835910 TF-232345 TF-571462
GO:0031497: P: chromatin assembly: 7.21002472134454e-08 (537,5,22,5) (57) Genes: G-571057 G-593861 G-655445 G-835910 TF-232345 TF-571462
GO:0000786: C: nucleosome: 7.21002472134454e-08 (537,5,22,5) (57) Genes: G-571057 G-593861 G-655445 G-835910 TF-232345 TF-571462
GO:0006333: P: chromatin assembly or disassembly: 9.21280936616831e-08 (537,5,23,5) (57) Genes: G-571057 G-593861 G-655445 G-835910 TF-232345 TF-571462
GO:0006323: P: DNA packaging: 1.16372328835786e-07 (537,5,24,5) (57) Genes: G-571057 G-593861 G-655445 G-835910 TF-232345 TF-571462
GO:0032993: C: protein-DNA complex: 1.16372328835786e-07 (537,5,24,5) (57) Genes: G-571057 G-593861 G-655445 G-835910 TF-232345 TF-571462
GO:0065004: P: protein-DNA complex assembly: 1.16372328835786e-07 (537,5,24,5) (57) Genes: G-571057 G-593861 G-655445 G-835910 TF-232345 TF-571462
GO:0000785: C: chromatin: 1.45465411044706e-07 (537,5,25,5) (57) Genes: G-571057 G-593861 G-655445 G-835910 TF-232345 TF-571462
GO:0071103: P: DNA conformation change: 1.80100032721933e-07 (537,5,26,5) (57) Genes: G-571057 G-593861 G-655445 G-835910 TF-232345 TF-571462
GO:0006325: P: chromatin organization: 2.69082262327723e-07 (537,5,28,5) (57) Genes: G-571057 G-593861 G-655445 G-835910 TF-232345 TF-571462
GO:0051276: P: chromosome organization: 3.25141066979586e-07 (537,5,29,5) (57) Genes: G-571057 G-593861 G-655445 G-835910 TF-232345 TF-571462
GO:0044427: C: chromosomal part: 3.90169280375086e-07 (537,5,30,5) (57) Genes: G-571057 G-593861 G-655445 G-835910 TF-232345 TF-571462
GO:0005694: C: chromosome: 1.37427440900929e-06 (537,5,38,5) (57) Genes: G-571057 G-593861 G-655445 G-835910 TF-232345 TF-571462
GO:0034622: P: cellular macromolecular complex assembly: 1.37427440900929e-06 (537,5,38,5) (57) Genes: G-571057 G-593861 G-641120 G-655445 G-835910 TF-232345 TF-571462
GO:0005730: C: nucleolus: 1.80156981348991e-06 (537,5,40,5) (57) Genes: G-571057 G-593861 G-641120 G-655445 G-825854 G-835910 TF-232345 TF-571462
GO:0034621: P: cellular macromolecular complex subunit organization: 1.80156981348991e-06 (537,5,40,5) (57) Genes: G-571057 G-593861 G-641120 G-655445 G-835910 TF-232345 TF-571462
GO:0065003: P: macromolecular complex assembly: 2.97339732953058e-06 (537,5,44,5) (57) Genes: G-571057 G-593861 G-641120 G-655445 G-835910 TF-232345 TF-571462
GO:0043933: P: macromolecular complex subunit organization: 3.75300760495385e-06 (537,5,46,5) (57) Genes: G-571057 G-593861 G-641120 G-655445 G-835910 TF-232345 TF-571462
GO:0031981: C: nuclear lumen: 6.43152603931213e-06 (537,5,51,5) (57) Genes: G-571057 G-593861 G-641120 G-655445 G-825854 G-835910 TF-232345 TF-571462
GO:0022607: P: cellular component assembly: 7.85695290973261e-06 (537,5,53,5) (57) Genes: G-571057 G-593861 G-641120 G-655445 G-835910 TF-232345 TF-571462
GO:0044428: C: nuclear part: 7.85695290973261e-06 (537,5,53,5) (57) Genes: G-571057 G-593861 G-641120 G-655445 G-825854 G-835910 TF-232345 TF-571462
GO:0070013: C: intracellular organelle lumen: 1.25454430151536e-05 (537,5,58,5) (57) Genes: G-571057 G-593861 G-641120 G-655445 G-825854 G-835910 TF-232345 TF-571462
GO:0043233: C: organelle lumen: 1.25454430151536e-05 (537,5,58,5) (57) Genes: G-571057 G-593861 G-641120 G-655445 G-825854 G-835910 TF-232345 TF-571462
GO:0006996: P: organelle organization: 3.0770090590543e-05 (537,5,69,5) (57) Genes: G-571057 G-593861 G-655445 G-835910 TF-232345 TF-571462
GO:0003677: F: DNA binding: 3.56474009871889e-05 (537,5,71,5) (57) Genes: G-571057 G-593861 G-655445 G-835910 TF-232345 TF-571462 TF-830919
GO:0043228: C: non-membrane-bounded organelle: 0.000277462679897905 (537,5,106,5) (57) Genes: G-571057 G-580797 G-593861 G-641120 G-655445 G-825854 G-835910 TF-232345 TF-571462
GO:0043232: C: intracellular non-membrane-bounded organelle: 0.000277462679897905 (537,5,106,5) (57) Genes: G-571057 G-580797 G-593861 G-641120 G-655445 G-825854 G-835910 TF-232345 TF-571462
GO:0003676: F: nucleic acid binding: 0.000696064928496575 (537,5,127,5) (57) Genes: G-571057 G-593861 G-655445 G-835910 TF-232345 TF-571462 TF-830919
GO:0005634: C: nucleus: 0.00102279444326757 (537,5,137,5) (57) Genes: G-227384 G-571057 G-593861 G-641120 G-655445 G-825854 G-835910 TF-232345 TF-571462 TF-830919
GO:0009292: P: genetic transfer: 0.00102675484791319 (537,5,6,2) (57) Genes: TF-232345 TF-571462
GO:0009294: P: DNA mediated transformation: 0.00102675484791319 (537,5,6,2) (57) Genes: TF-232345 TF-571462
GO:0009415: P: response to water: 0.0013216984372719 (537,5,29,3) (57) Genes: G-571057 G-593861 G-655445 G-835910
GO:0009414: P: response to water deprivation: 0.0013216984372719 (537,5,29,3) (57) Genes: G-571057 G-593861 G-655445 G-835910
GO:0005773: C: vacuole: 0.00161645084351514 (537,5,31,3) (57) Genes: G-593861 G-655445 G-835910
GO:0006950: P: response to stress: 0.00270630771608582 (537,5,166,5) (57) Genes: G-227384 G-571057 G-580797 G-593861 G-641120 G-655445 G-835910 TF-232345 TF-571462
GO:0009611: P: response to wounding: 0.0111436592406034 (537,5,19,2) (57) Genes: TF-232345 TF-571462
Significance threshold KEGG: 0 (FDR)

IF OS~ATGCAAAT_motif AND PS~I-box THEN G-718770-0.6
G-718770-0.6: 0.000015 (562,26,21,7)
Cov-neg: G-197763 G-282545 G-288961 G-290157 G-555320 G-569972 G-570826 G-653119 G-668020 G-706715 G-723778 G-750752 G-752731 G-813709 G-820850 G-824462 G-828416 G-832984 TF-836808
Cov-pos: G-549486 G-560859 G-572955 G-581217 G-647016 G-654065 G-718770

Significance threshold GO: 0.000644253578536587 (FDR)
GO:0009532: C: plastid stroma: 0.000644253578536587 (537,7,120,6) (196) Genes: G-197763 G-549486 G-560859 G-572955 G-581217 G-647016 G-706715 G-718770 G-820850
Significance threshold KEGG: 0 (FDR)

IF LE~circadian AND SA~Box_II THEN G-819059-0.5
G-819059-0.5: 0.000035 (562,21,20,6)
Cov-neg: G-173755 G-410396 G-557360 G-581162 G-643350 G-671325 G-715371 G-737939 G-784099 G-815393 G-817211 G-817545 G-817745 G-835035 TF-710397
Cov-pos: G-414955 G-568456 G-572020 G-709740 G-819059 G-821685
Significance threshold GO: 0.000619143763715988 (FDR)
GO:0009769: P: photosynthesis, light harvesting in photosystem II: 0.000619143763715988 (537,6,4,2) (128) Genes: G-568456 G-709740
Significance threshold KEGG: 0 (FDR)

IF MA0044.1_HMG-1 AND OS~MPE THEN TF-834586-0.5
TF-834586-0.5: 0.001525 (562,41,26,7)
Cov-neg: G-179495 G-249644 G-258051 G-267999 G-417459 G-549292 G-560859 G-564642 G-568201 G-574284 G-580161 G-641120 G-641273 G-642306 G-653119 G-720677 G-726819 G-728017 G-763593 G-798453 G-815557 G-816237 G-817211 G-824231 G-826253 G-831725 G-831727 G-832630 G-832825 G-833331 G-834077 G-837476 TF-209269 TF-566736
Cov-pos: G-206841 G-242343 G-279999 G-576481 G-597236 G-725763 TF-834586
Significance threshold GO: 0 (FDR)
Significance threshold KEGG: 0 (FDR)

IF PH~CCGTCC-box AND PS~ATCC-motif AND ZM~Unnamed__2 THEN G-725114-0.75
G-725114-0.75: 1.429211e-07 (562,32,7,6)
Cov-neg: G-179495 G-227384 G-234689 G-245912 G-249644 G-279999 G-293436 G-296649 G-416054 G-557360 G-564642 G-571057 G-573011 G-576481 G-654077 G-680311 G-706393 G-722365 G-734671 G-819059 G-821599 G-823815 G-827624 G-836585 TF-571462 TF-710397
Cov-pos: G-553020 G-655455 G-725114 G-725127 G-747465 G-833496
Significance threshold GO: 0.0279766057532186 (FDR)
GO:0034728: P: nucleosome organization: 2.30395526810035e-09 (537,6,22,6) (54) Genes: G-553020 G-564642 G-571057 G-655455 G-725114 G-725127 G-747465 G-833496 TF-571462
GO:0006334: P: nucleosome assembly: 2.30395526810035e-09 (537,6,22,6) (54) Genes: G-553020 G-564642 G-571057 G-655455 G-725114 G-725127 G-747465 G-833496 TF-571462
GO:0031497: P: chromatin assembly: 2.30395526810035e-09 (537,6,22,6) (54) Genes: G-553020 G-564642 G-571057 G-655455 G-725114 G-725127 G-747465 G-833496 TF-571462
GO:0000786: C: nucleosome: 2.30395526810035e-09 (537,6,22,6) (54) Genes: G-553020 G-564642 G-571057 G-655455 G-725114 G-725127 G-747465 G-833496 TF-571462
GO:0006333: P: chromatin assembly or disassembly: 3.11711595095771e-09 (537,6,23,6) (54) Genes: G-553020 G-564642 G-571057 G-655455 G-725114 G-725127 G-747465 G-833496 TF-571462
GO:0006323: P: DNA packaging: 4.1561546012791e-09 (537,6,24,6) (54) Genes: G-553020 G-564642 G-571057 G-655455 G-706393 G-725114 G-725127 G-747465 G-833496 TF-571462
GO:0032993: C: protein-DNA complex: 4.1561546012791e-09 (537,6,24,6) (54) Genes: G-553020 G-564642 G-571057 G-655455 G-725114 G-725127 G-747465 G-833496 TF-571462
GO:0065004: P: protein-DNA complex assembly: 4.1561546012791e-09 (537,6,24,6) (54) Genes: G-553020 G-564642 G-571057 G-655455 G-725114 G-725127 G-747465 G-833496 TF-571462
GO:0000785: C: chromatin: 5.46862447536598e-09 (537,6,25,6) (54) Genes: G-553020 G-564642 G-571057 G-655455 G-725114 G-725127 G-747465 G-833496 TF-571462
GO:0071103: P: DNA conformation change: 7.10921181797639e-09 (537,6,26,6) (54) Genes: G-553020 G-564642 G-571057 G-655455 G-706393 G-725114 G-725127 G-747465 G-833496 TF-571462
GO:0006325: P: chromatin organization: 1.16332557021402e-08 (537,6,28,6) (54) Genes: G-553020 G-564642 G-571057 G-655455 G-706393 G-725114 G-725127 G-747465 G-833496 TF-571462
GO:0051276: P: chromosome organization: 1.46680180592433e-08 (537,6,29,6) (54) Genes: G-553020 G-564642 G-571057 G-655455 G-706393 G-725114 G-725127 G-747465 G-833496 TF-571462
GO:0044427: C: chromosomal part: 1.83350225740341e-08 (537,6,30,6) (54) Genes: G-553020 G-564642 G-571057 G-655455 G-725114 G-725127 G-747465 G-833496 TF-571462
GO:0005694: C: chromosome: 8.52463449196378e-08 (537,6,38,6) (54) Genes: G-553020 G-564642 G-571057 G-655455 G-706393 G-725114 G-725127 G-747465 G-833496 TF-571462
GO:0034622: P: cellular macromolecular complex assembly: 8.52463449196378e-08 (537,6,38,6) (54) Genes: G-553020 G-564642 G-571057 G-655455 G-725114 G-725127 G-747465 G-833496 TF-571462

GO:0034621: P: cellular macromolecular complex subunit organization: 1.18524329834883e-07 (537,6,40,6) (54) Genes: G-553020 G-564642 G-571057 G-655455 G-725114 G-725127 G-747465 G-833496 TF-571462
GO:0065003: P: macromolecular complex assembly: 2.17974616262567e-07 (537,6,44,6) (54) Genes: G-553020 G-564642 G-571057 G-655455 G-725114 G-725127 G-747465 G-833496 TF-571462
GO:0043933: P: macromolecular complex subunit organization: 2.89235548502116e-07 (537,6,46,6) (54) Genes: G-553020 G-564642 G-571057 G-655455 G-725114 G-725127 G-747465 G-833496 TF-571462
GO:0022607: P: cellular component assembly: 7.08898006893317e-07 (537,6,53,6) (54) Genes: G-553020 G-564642 G-571057 G-655455 G-725114 G-725127 G-747465 G-833496 TF-571462
GO:0006996: P: organelle organization: 3.70166503345148e-06 (537,6,69,6) (54) Genes: G-245912 G-553020 G-564642 G-571057 G-655455 G-706393 G-725114 G-725127 G-747465 G-833496 TF-571462
GO:0003677: F: DNA binding: 4.42242192698108e-06 (537,6,71,6) (54) Genes: G-553020 G-564642 G-571057 G-655455 G-706393 G-725114 G-725127 G-734671 G-747465 G-833496 TF-571462 TF-710397
GO:0005730: C: nucleolus: 1.02167972317589e-05 (537,6,40,5) (54) Genes: G-553020 G-564642 G-571057 G-655455 G-706393 G-725114 G-725127 G-833496 TF-571462
GO:0031981: C: nuclear lumen: 3.58086092640189e-05 (537,6,51,5) (54) Genes: G-553020 G-564642 G-571057 G-655455 G-706393 G-725114 G-725127 G-833496 TF-571462
GO:0044428: C: nuclear part: 4.35972274239385e-05 (537,6,53,5) (54) Genes: G-553020 G-564642 G-571057 G-655455 G-706393 G-725114 G-725127 G-833496 TF-571462
GO:0043228: C: non-membrane-bounded organelle: 5.26761854692968e-05 (537,6,106,6) (54) Genes: G-553020 G-564642 G-571057 G-655455 G-706393 G-725114 G-725127 G-747465 G-833496 TF-571462
GO:0043232: C: intracellular non-membrane-bounded organelle: 5.26761854692968e-05 (537,6,106,6) (54) Genes: G-553020 G-564642 G-571057 G-655455 G-706393 G-725114 G-725127 G-747465 G-833496 TF-571462
GO:0070013: C: intracellular organelle lumen: 6.90235182431647e-05 (537,6,58,5) (54) Genes: G-553020 G-564642 G-571057 G-655455 G-706393 G-725114 G-725127 G-833496 TF-571462
GO:0043233: C: organelle lumen: 6.90235182431647e-05 (537,6,58,5) (54) Genes: G-553020 G-564642 G-571057 G-655455 G-706393 G-725114 G-725127 G-833496 TF-571462
GO:0003676: F: nucleic acid binding: 0.000159623912174004 (537,6,127,6) (54) Genes: G-553020 G-564642 G-571057 G-576481 G-655455 G-706393 G-725114 G-725127 G-734671 G-747465 G-833496 TF-571462 TF-710397
GO:0005634: C: nucleus: 0.000253776064870657 (537,6,137,6) (54) Genes: G-227384 G-249644 G-553020 G-564642 G-571057 G-655455 G-706393 G-725114 G-725127 G-747465 G-833496 TF-571462 TF-710397
GO:0044446: C: intracellular organelle part: 0.0279766057532186 (537,6,297,6) (54) Genes: G-227384 G-245912 G-293436 G-296649 G-416054 G-553020 G-564642 G-571057 G-576481 G-654077 G-655455 G-680311 G-706393 G-725114 G-725127 G-734671 G-747465 G-819059 G-827624 G-833496 TF-571462
Significance threshold KEGG: 0 (FDR)

IF GM~Unnamed__5 AND M00182-P$GBP_Q6 AND M00443-P$O2_Q2 THEN G-204897-0.55
G-204897-0.55: 0.000013 (562,29,8,5)
Cov-neg: G-267999 G-279999 G-288961 G-417459 G-421814 G-552732 G-568456 G-571855 G-572618 G-641273 G-652151 G-677905 G-706284 G-721810 G-731296 G-732598 G-743149 G-743910 G-746197 G-752731 G-784099 G-828771 G-830089 TF-740041
Cov-pos: G-204897 G-553020 G-670620 G-715371 G-725114
Significance threshold GO: 0.0164031620112056 (FDR)
GO:0034728: P: nucleosome organization: 1.03864411680404e-05 (537,5,22,4) (99) Genes: G-553020 G-670620 G-715371 G-725114
GO:0006334: P: nucleosome assembly: 1.03864411680404e-05 (537,5,22,4) (99) Genes: G-553020 G-670620 G-715371 G-725114
GO:0031497: P: chromatin assembly: 1.03864411680404e-05 (537,5,22,4) (99) Genes: G-553020 G-670620 G-715371 G-725114
GO:0000786: C: nucleosome: 1.03864411680404e-05 (537,5,22,4) (99) Genes: G-553020 G-670620 G-715371 G-725114
GO:0006333: P: chromatin assembly or disassembly: 1.25536649731715e-05 (537,5,23,4) (99) Genes: G-553020 G-670620 G-715371 G-725114
GO:0006323: P: DNA packaging: 1.50411235020343e-05 (537,5,24,4) (99) Genes: G-553020 G-670620 G-715371 G-725114
GO:0032993: C: protein-DNA complex: 1.50411235020343e-05 (537,5,24,4) (99) Genes: G-553020 G-572618 G-670620 G-715371 G-725114
GO:0065004: P: protein-DNA complex assembly: 1.50411235020343e-05 (537,5,24,4) (99) Genes: G-553020 G-572618 G-670620 G-715371 G-725114
GO:0000785: C: chromatin: 1.78783917098395e-05 (537,5,25,4) (99) Genes: G-553020 G-670620 G-715371 G-725114
GO:0071103: P: DNA conformation change: 2.10962629238426e-05 (537,5,26,4) (99) Genes: G-553020 G-572618 G-670620 G-715371 G-725114
GO:0006325: P: chromatin organization: 2.88030138300234e-05 (537,5,28,4) (99) Genes: G-553020 G-670620 G-715371 G-725114
GO:0051276: P: chromosome organization: 3.33594734720947e-05 (537,5,29,4) (99) Genes: G-553020 G-670620 G-715371 G-725114
GO:0044427: C: chromosomal part: 3.84316741169548e-05 (537,5,30,4) (99) Genes: G-553020 G-572618 G-670620 G-715371 G-725114
GO:0005694: C: chromosome: 0.000102221764128962 (537,5,38,4) (99) Genes: G-553020 G-572618 G-670620 G-715371 G-725114
GO:0034622: P: cellular macromolecular complex assembly: 0.000102221764128962 (537,5,38,4) (99) Genes: G-553020 G-572618 G-670620 G-715371 G-725114
GO:0034621: P: cellular macromolecular complex subunit organization: 0.000126159930550318 (537,5,40,4) (99) Genes: G-553020 G-572618 G-670620 G-715371 G-725114
GO:0065003: P: macromolecular complex assembly: 0.000186209007761808 (537,5,44,4) (99) Genes: G-553020 G-572618 G-670620 G-715371 G-725114 G-752731

GO:0043933: P: macromolecular complex subunit organization: 0.000223125237846985 (537,5,46,4) (99) Genes: G-553020 G-572618 G-670620 G-715371 G-725114 G-752731
GO:0022607: P: cellular component assembly: 0.000395894219064235 (537,5,53,4) (99) Genes: G-553020 G-572618 G-670620 G-715371 G-725114 G-752731
GO:0006996: P: organelle organization: 0.00113849335185009 (537,5,69,4) (99) Genes: G-553020 G-670620 G-677905 G-715371 G-721810 G-725114
GO:0003677: F: DNA binding: 0.00127532567412383 (537,5,71,4) (99) Genes: G-267999 G-553020 G-572618 G-670620 G-715371 G-725114
GO:0005730: C: nucleolus: 0.00346030841300343 (537,5,40,3) (99) Genes: G-267999 G-553020 G-670620 G-725114
GO:0043228: C: non-membrane-bounded organelle: 0.00613954184832901 (537,5,106,4) (99) Genes: G-267999 G-553020 G-572618 G-670620 G-715371 G-725114
GO:0043232: C: intracellular non-membrane-bounded organelle: 0.00613954184832901 (537,5,106,4) (99) Genes: G-267999 G-553020 G-572618 G-670620 G-715371 G-725114
GO:0031981: C: nuclear lumen: 0.00705870245888177 (537,5,51,3) (99) Genes: G-267999 G-553020 G-572618 G-670620 G-725114
GO:0044428: C: nuclear part: 0.00789277420116459 (537,5,53,3) (99) Genes: G-267999 G-553020 G-572618 G-670620 G-725114
GO:0070013: C: intracellular organelle lumen: 0.010240439624002 (537,5,58,3) (99) Genes: G-267999 G-553020 G-572618 G-670620 G-725114
GO:0043233: C: organelle lumen: 0.010240439624002 (537,5,58,3) (99) Genes: G-267999 G-553020 G-572618 G-670620 G-725114
GO:0003676: F: nucleic acid binding: 0.0122971470700928 (537,5,127,4) (99) Genes: G-267999 G-553020 G-572618 G-670620 G-715371 G-725114
GO:0005634: C: nucleus: 0.0164031620112056 (537,5,137,4) (99) Genes: G-267999 G-421814 G-553020 G-572618 G-670620 G-677905 G-715371 G-725114 G-828771
TF-740041
Significance threshold KEGG: 0 (FDR)

GO   SIGNIFICANT: 0.605263157894737 (23/38)
KEGG SIGNIFICANT: 0.695652173913043 (16/23)

TOT   SIGNIFICANT: 0.710526315789474 (27/38)
